# Evaluation of a low-cost staining method for improved visualization of sweet potato whitefly (*Bemisia tabaci*) eggs on multiple crop plant species

**DOI:** 10.1186/s13007-024-01209-z

**Published:** 2024-05-23

**Authors:** Benjamin van Raalte, Kristal Watrous, Miguel Lujan, Ricky Le, Penglin Sun, Benjamin Ellis, Kerry E. Mauck

**Affiliations:** 1 Department of Entomology, University of California, Riverside, 165 Entomology Bldg., Citrus Drive, Riverside, CA 92521 USA; 2 Department of Ecology and Evolutionary Biology, University of California, Irvine, 321 Steinhaus Hall, Irvine, CA 92697 USA; 3 Department of Statistics, University of California, Riverside, 900 University Ave., Olmsted Hall 1337, Riverside, CA 92521 USA

**Keywords:** Host plant resistance, Phenotyping, Cassava, Cowpea, Melon, Sweet potato, Tomato

## Abstract

**Background:**

The sweet potato whitefly (*Bemisia tabaci*) is a globally important insect pest that damages crops through direct feeding and by transmitting viruses. Current *B. tabaci* management revolves around the use of insecticides, which are economically and environmentally costly. Host plant resistance is a sustainable option to reduce the impact of whiteflies, but progress in deploying resistance in crops has been slow. A major obstacle is the high cost and low throughput of screening plants for *B. tabaci* resistance. Oviposition rate is a popular metric for host plant resistance to *B. tabaci* because it does not require tracking insect development through the entire life cycle, but accurate quantification is still limited by difficulties in observing *B. tabaci* eggs, which are microscopic and translucent. The goal of our study was to improve quantification of *B. tabaci* eggs on several important crop species: cassava, cowpea, melon, sweet potato and tomato.

**Results:**

We tested a selective staining process originally developed for leafhopper eggs: submerging the leaves in McBryde’s stain (acetic acid, ethanol, 0.2% aqueous acid Fuchsin, water; 20:19:2:1) for three days, followed by clearing under heat and pressure for 15 min in clearing solution (LGW; lactic acid, glycerol, water; 17:20:23). With a less experienced individual counting the eggs, *B. tabaci* egg counts increased after staining across all five crops. With a more experienced counter, egg counts increased after staining on melons, tomatoes, and cowpeas. For all five crops, there was significantly greater agreement on egg counts across the two counting individuals after the staining process. The staining method worked particularly well on melon, where egg counts universally increased after staining for both counting individuals.

**Conclusions:**

Selective staining aids visualization of *B. tabaci* eggs across multiple crop plants, particularly species where leaf morphological features obscure eggs, such as melons and tomatoes. This method is broadly applicable to research questions requiring accurate quantification of *B. tabaci* eggs, including phenotyping for *B. tabaci* resistance.

**Supplementary Information:**

The online version contains supplementary material available at 10.1186/s13007-024-01209-z.

## Introduction

The sweet potato whitefly (*Bemisia tabaci*) (Hemiptera: Aleyrodidae) is an agricultural pest of global significance with a wide host range that includes many crop species [1–3]. At high population densities, *B. tabaci* cause damage to crops through several mechanisms: the removal of phloem sap and direct damage to leaf tissues, inducing disorders from the injection of saliva into the phloem, and excreting honeydew that can cause secondary infections and interfere with photosynthesis [4–6]. In zucchini for example, yield losses of 25–100% have been reported from direct damage under high levels of *B. tabaci* infestation [7]. *B. tabaci* are also virus vectors in many crops.

Host plant resistance can be a valuable component of integrated pest management for *B. tabaci* [8–10]. Host plant resistance poses fewer risks to the environment and worker health than insecticides, and is less complex to implement when compared to many other lower risk cultural control tactics [11]. Host plant resistance is typically divided into three functional categories, antibiosis: negative effects on the insect biology measured with no-choice assays, antixenosis: host finding or ‘preference’ measured with choice assays, and tolerance: ability to sustain insect damage with reduced loss of yield or quality [12]. Choice assays are when individual insects have access to multiple host genotypes, and analyses compare where the insects ‘choose’ to feed, settle, or reproduce. No-choice assays are when the insects are constrained to a single host plant genotype and their development is compared to other insects confined to a different host genotype.

Resistance to *B. tabaci* has been identified in many important crop families including: Brassicaceae, Cucurbitaceae, Euphorbiaceae, Fabaceae, Malvaceae, Pedaliaceae, and Solanaceae [8, 13]. This resistance may arise from a variety of traits and mechanisms that are largely not well understood. In all of these cases, the identified resistance traits confer partial resistance, with a measured improvement in antibiosis, antixenosis or tolerance for the ‘resistant’ plant genotype compared to a ‘susceptible’ genotype. None of the resistant varieties in any of these crops completely prevent B. tabaci feeding or development. Despite promising research published over the past two decades identifying plant germplasm with *B. tabaci* resistance [14–20], we do not know of any *B. tabaci* resistant varieties that are commercially available for crop producers.

One reason why B. tabaci resistance traits have not yet been deployed in commercial varieties is a lack of time and labor efficient methods to phenotype for host plant resistance. Traditional screening methods for *B. tabaci* resistance in lab and greenhouse settings involve allowing *B. tabaci* to develop on the plant genotypes of interest and measuring many insect development traits, such as: oviposition rate, rate of development of each life stage, adult longevity, and overall population dynamics [16, 21–25]. Another common technique to detect *B. tabaci* resistance is to expose the plants to whiteflies for a short period, then measure the adult emergence of one generation by counting 4th instar nymph exuviae [23, 26–28]. These methods require considerable amounts of time, labor, space, and skilled investigators capable of identifying and tracking tiny whiteflies through many life stages, greatly reducing the throughput of host plant resistance phenotyping.

Field trials to screen for *B. tabaci* resistance have been conducted in multiple crop species [29–33]. These trials have typically compared the number of whiteflies per leaf or per leaf area on different plant genotypes to assess the level of host plant resistance, with some studies counting just adults and others counting eggs, nymphs and adults. Field trials address some of the limitations of lab and greenhouse experiments in that they are easier to scale to screen large amounts of germplasm, and account for more real world factors (weather, insect predators, etc.), but still have several issues. Depending on the latitude of the field sites, it may only be possible to run one experiment per year. It can also be difficult to ensure consistent *B. tabaci* pressure across trials [31], further slowing research progress.

Oviposition tracking is one viable alternative to the problematic *B. tabaci* resistance phenotyping approaches mentioned above. Prior studies show that low rates of oviposition (number of eggs laid per female per unit time) strongly correlate with other developmental parameters used to identify resistant germplasm, including many parameters that are time and labor-intensive to measure. Oviposition can be used as a response variable to measure either antibiosis or antixenosis, depending on if it is recorded in respectively, a no-choice or choice assay. Rodríguez-Álvarez et al. [17] detected host plant resistance to *B. tabaci* in tomato using a six-day no-choice oviposition assay. They found that resistance identified through oviposition tracking was well supported by development parameters measured in both no-choice and choice assays. Coelho et al. [19] conducted choice testing on 32 melon genotypes and selected seven cultivars representing the full range of *B. tabaci* preference for entry into a seven-day no-choice oviposition assay. They detected the strongest resistance in the same melon genotype (cv. ‘Neve’) as was expected from the choice assay results. This finding was also supported by *B. tabaci* development parameters measured in other experiments. In a different study screening 12 cucumber genotypes for resistance to *B. tabaci*, significant differences in oviposition were found from a seven-day no-choice assay and those results were strongly correlated to “degree of colonization”, but not as strongly to other measured development parameters [16]. In a group of ten soybean genotypes, differences in oviposition from a three-day no-choice assay were used to identify a single putative resistant genotype (‘IAC-17’), and other experiments tracking nymph life parameters also found slightly slowed *B. tabaci* development on that genotype – along with several other soybean genotypes included in the study [18]. A more recent study in soybean identified four genotypes as resistant, with two of those four selected due to low oviposition values from a three day no-choice assay [20].

These studies indicate that tracking no-choice oviposition is a viable phenotyping approach to identify *B. tabaci*-resistant plant material. While this approach has significantly higher throughput and is less labor-intensive than developmental assays, there are still challenges. *B. tabaci* eggs are mostly translucent and very small (200 μm at the longest axis) [34]. As a result, they are difficult to quantify reliably on the wide variety of different leaf surface features associated with *B. tabaci* hosts. Improving egg visualization would address these limitations and facilitate wider use of no-choice oviposition as a metric for host plant resistance phenotyping.

Egg visualization challenges can be overcome with high-resolution microscopy, but the costs associated with these instruments are out of reach for most breeding programs [35]. To address this issue and improve the viability of oviposition tracking as a phenotyping tool, we adapted and tested a low-cost, selective staining approach for egg visualization and counting across five crop species that experience significant losses due to *B. tabaci* feeding. *B. tabaci* are a complex of at least 24 morphologically indistinguishable species [36]. We will consider these species together in the context of this paper because egg visualization is a potential challenge across all species in the complex, and there is not enough published information about *B. tabaci* eggs to elucidate any differences in egg morphology or chemistry across the species complex. The staining technique identified was originally used to stain fungal hyphae [37], then later adapted to stain leafhopper eggs and salivary sheaths [38]. The objective of our study was to: (a) determine if this “McBryde’s stain” could be used to selectively stain *B. tabaci* eggs, (b) reduce cost and complexity of the original method, and (c) explore the range of crop species for which this method might be useful.

Developing this tool to improve the quantification of *B. tabaci* oviposition rate will facilitate higher throughput and lower cost phenotyping for host plant resistance to this damaging pest species. Better phenotyping methods allow breeders to screen more plant germplasm in a single trial, leading to the identification of new resistance genes and more rapid progression of germplasm through the breeding process. More accurate phenotyping can detect smaller differences in a trait, which is particularly useful when breeding or developing markers for complex quantitative traits controlled by many minor genes [39]. Together these improvements should facilitate the development and deployment of more crop varieties with *B. tabaci* resistance.

## Methods

### Plant materials

We used the commercial muskmelon (*Cucumis melo* L.) varieties ‘Top Mark’ (USDA GRIN: NSL 30032) (Everwilde Farms, Sand Creek, WI, USA), ‘Gold Crown’ (Syngenta, Hopkins, MN, USA) or accession TGR-1551 (PI 482420; 2017 internal increase by K. Mauck, originally sourced from melon breeder Dr. Jim McCreight, USDA-ARS). ‘Gold Crown’ melon seeds were treated with FarMore® F300 fungicide (active ingredients: mefenoxam, fludioxonil, and azoxystrobin; Syngenta, Hopkins, MN, USA). All other seeds used in this project were untreated. Cowpeas (*Vigna unguiculata* (L.) Walp.) used were the variety ‘Mississippi Silver’ (Willhite Seed Inc., Poolville, TX, USA). Tomato (*Solanum lycopersicum* L.) varieties used were ‘Moneymaker’ (LA2706) (Isla’s Garden Seeds, Eagle, ID, USA), and ‘Motelle’ (LA 2823) (2023 seed increase, original accession from the C.M. Rick Tomato Genetics Resource Center at UC Davis, Davis, CA, USA). Cassava (*Manihot esculenta* Crantz) seeds used were an unknown variety purchased from Trade Winds Fruit (Santa Rosa, CA). Sweet potato (*Ipomoea batatas* (L.) Lam.) cuttings used were cultivars ‘Beauregard’ (PI 566613) and ‘Bellevue’ provided by C. Scott Stoddard of University of California, Merced County Cooperative Extension [40]. Before shipment to the authors, sweet potato cuttings were produced in a commercial nursery using conventional methods for sweet potato cultivation.

For cassava, cowpea, melon, and tomato, all seeds were started in plastic growing cells with a volume of about 200 ml. Each cell was filled with 10:1:1 mix of UC Soil Mix 3 (UC Riverside Agricultural Operations, Riverside, CA, USA) to perlite (Therm-O-Rock West, Chandler, AZ, USA) to coarse vermiculite (PVP Industries, North Bloomfield, OH, USA) (henceforth ‘growing media’), and 2.5 g of slow release fertilizer pellets (Osmocote® Plus Indoor & Outdoor 15-9-12 six month release, ICL Group, St. Louis, MO, USA) (henceforth ‘Osmocote®’) per cell [41]. Two seeds were added per pot; melon and cassava seeds were covered under 1 cm of growing media, cowpea seeds were covered under 1.5 cm of growing media, and tomato seeds were covered under 0.25 cm of growing media. Plants were kept in a lab growth chamber (Percival E-36HO, Percival Scientific, Perry, IA, USA) set at 25°C and 24 hours light. After 8–10 days seeds were thinned to leave only a single plant per pot. Melons and cowpea plants were transplanted after 10–15 days, cassava plants were transplanted after 34 days, and tomato plants were transplanted after 27 days. When transplanted, all crops were moved into larger pots of about 600 ml, retaining all original growing media and containing about 400 ml of new growing media with 2.5 g of additional Osmocote®. After transplanting, plants were moved from the growth chamber into a greenhouse in University of California, Riverside’s Insectary and Quarantine facility (33° 58’ 13.2” N, 117° 19’ 29.5” W; Riverside, CA, USA) with temperature held at 27 (± 2)°C. In addition to the natural light, to supply a consistent photoperiod in the greenhouse, 16 h of supplemental light was provided using 1500 W LED grow lights (YGROW, Shenzhen Yanggu Lighting Co., Ltd., Shenzhen, China; purchased on Amazon.com). Once in the greenhouse, all plants (except cassava) were trained vertically onto 3 mm 6 ply jute twine. Cassava has an upright growth habit so no vertical training was required. Across all crops, lateral shoot growth was removed regularly to manage plant size within the limited greenhouse space.

Sweet potatoes were rooted by inserting the cuttings about 10 cm into 600 ml pots filled with growing media and five grams of Osmocote®. Pots were kept moist in a growth chamber until roots were visible growing out of the bottom of the pots, then the plants were moved to the greenhouse and trained vertically. Due to the presence of citrus mealy bugs (*Planococcus citri*) on the sweet potato plants, twelve days before use in *B. tabaci* exposure experiments, the lower sections of foliage were treated with insecticidal soap (active ingredient: potassium salts of fatty acids; Safer® Brand Insect Killing Soap, www.saferbrand.com) diluted according to the label instructions. Care was taken during the insecticidal soap application to avoid treating any upper leaves that would later be used for the *B. tabaci* exposure assays.

### No-choice containment cages

The clip cages used are a modified version of the MacGillivray and Anderson clip cage [42], and are also similar in design to clip cages used by other authors [43]. Clip cages are constructed out of two Corning™ Falcon™ 50 ml conical centrifuge tubes (Corning Incorporated, NY, NY, USA) with the bottom of the tubes cut off and separated by fine mesh (Figure S1 A). This mesh allows air flow to the leaf and the caged insects. The leaf clips interface with the leaf surface on the insect cage side with a modified Falcon™ tube cap with a 24 mm hole cut into it, and that is pressed against a 35 mm plastic disc on the opposite side of the leaf. 4.76 mm thick polyurethane foam (76 Flex-Foam™, Pellon, Clearwater, FL, USA) is affixed to both sides these plastic parts to provide a good seal to prevent insect escape without damaging the leaf (Figure S1 B). An aluminum hair sectioning clip (Goody Products, Atlanta, GA, USA) holds the disc and cap pieces together and applies pressure to seal the foam against the leaf (Figure S1 C). Further details on clip cage design and construction is provided in Supplemental Methods and in Figure S1.

### Whiteflies

All whiteflies used were *Bemisia tabaci* MEAM1 from lab lines Mac1 and Mac2 and were positive for the *Rickettsia* mutualist bacteria [44]. The *B. tabaci* line used was always consistent within a single exposure experiment. *B. tabaci* colonies were maintained on cowpeas. Colonies were maintained in a 166 micron (about 150 US mesh size) mesh bag supported by a metal cage, and that entire assembly was kept inside a mesh and plastic insect rearing tent (BugDorm-2S120, MegaView Science Co., Ltd., Taichung, Taiwan). Colonies were kept at 25.5 (± 2)°C and relative humidity of 30–50%.

To prepare for exposure experiments, individual whiteflies were aspirated out of the *B. tabaci* colonies into small 11.5 ml tubes. Tubes containing the whiteflies were sorted by sex under a dissecting scope. Only the female whiteflies were used for these experiments. Five females were then loaded into each containment cage using an inline aspiration attachment (Figure S1 D).

### *Bemisia tabaci* exposure assays

Plant ages for *B. tabaci* exposure were selected based on (a) when resistance has been previously reported in those crops, or (b) when the plants had at least four leaves large enough to accept a clip cage. Plant ages in days at the start of each *B. tabaci* oviposition assay are as follows: 60 for cassava (*n* = 8 plants; two clip cages were used per plant), 27 for cowpea (*n* = 15), 41 for the first melon assay (*n* = 16), 59 for the second melon assay (*n* = 15), 63 for the first tomato assay (*n* = 15), 97 for the second tomato assay (*n* = 15), 48 for sweet potato (*n* = 17) (Table S1). All no-choice *B. tabaci* exposure assays were conducted between June 4th and August 28th, 2023. All assays were conducted at the UCR Insectary and Quarantine greenhouse.

**Fig. 1:**
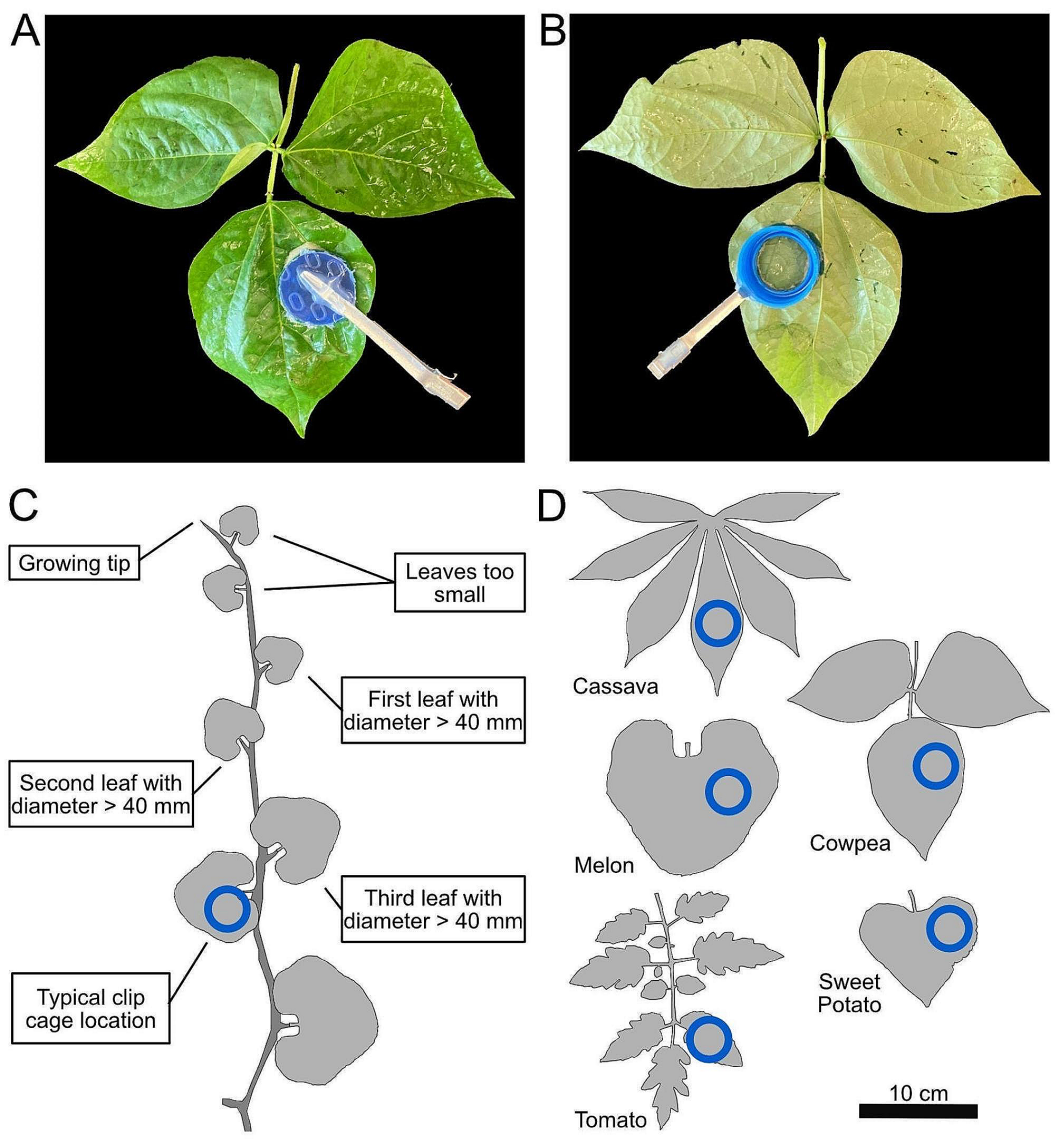
Positioning of clip cages on each crop. **A**. A top down view of leaf clip on a cowpea leaf. **B**. A bottom up view of a leaf clip on a cowpea leaf. **C.** Positions of cages on plants relative to apical growth, which was consistent across crops. A melon plant is used as an example. Leaf sizes are not to scale. The clip cage was always placed on the third or fourth most apical leaf able to accept a cage, which requires either a leaf (melon, sweet potato) or leaflet (cassava, cowpea, and tomato) diameter of at least 40 mm. **D**. Location where the clip cage was attached on representative leaves of each crop

Leaf age and position is known to affect *B. tabaci* oviposition, and was therefore kept consistent for these experiments [45]. To select the leaf for leaf clip attachment, we started at the new growing tip and moved down the stem to find the first suitable leaf, which must have an uninterrupted round surface with at least a diameter of 40 mm. The leaf clip was then attached three to four leaves further down the stem. This placement ensures that the leaves are fully expanded before cage attachment, while still being on a young enough leaf to allow screening of young plants. Exact desired leaf placement may vary depending on phenotyping goals. Details of placement position on each leaf, including the specific leaflet (cowpea, tomato) or leaf lobe (cassava), are shown in Fig. 1C. All cages were oriented so the underside of the leaves would be exposed to the whiteflies. Leaf clips were also attached to the training twine using a piece of labeling tape to support the weight of the cage and prevent damage to the leaf. After the leaf clips were attached to the plants, the filled containment tube devices were chilled at 4 °C for 10–15 min to slow down the whiteflies and prevent escape while attaching the containment cages to the leaf clips.

Starting one day after attaching the clip cages, and continuing for the duration of the no-choice exposures, *B. tabaci* adult survival was tracked by looking through the plastic sides and mesh bottom of the clip cage and counting all living whiteflies feeding on the leaf surface. This allows *B. tabaci* mortality to be accounted for when calculating oviposition rate. The *B. tabaci* survival data generated may be useful for some phenotyping applications, but survival data was not evaluated separately from oviposition for this study.

At the end of the oviposition exposure period (3–4 days), the leaves with attached cages were cut off the plants and moved out of the greenhouse to a 4 °C refrigerator. Leaves were chilled for 10–15 min to slow down whiteflies, then clip cages were taken off the leaf clips and whiteflies were aspirated off the leaves. Using a small paintbrush, a droplet of water was then placed at the center of the exposure area as a mark, and the leaf clip was removed. The leaf section exposed to the *B. tabaci* was then excised, using the droplet of water as a guide to center on the exposure area, by pressing a 38 mm round stainless steel cookie cutter (Sosohome, Goyang, South Korea) into the leaf and twisting slightly. A 50 mm x 17 mm glass petri dish (Eisco Scientific, Victor, NY, USA) was labeled with the plant number and any other identifying information and the excised leaf disc was then placed into the petri dish. To label the petri dishes, a small section of labeling tape is affixed to the side of the dish and label information is written in pencil. Using a pencil is important because spilled staining solution can remove ink from a pen or permanent marker.

### Egg counts and leaf imaging

After excising each leaf disc, the discs were moved to the lab and the eggs present on each disc were counted by two of the authors, Ben van Raalte (hereafter “counter one”) and Ricky Le (hereafter “counter two”). At the start of these experiments, counter one had about one year of experience working with *B. tabaci* and three years of general entomology experience, while counter two was new to entomology and to working with *B. tabaci*. Counts were made using a trinocular stereo microscope (SM4T, Amscope, Irvine, CA, USA) with 10X eyepieces and a 0.5X objective lens (Amscope WD165) for a total magnification range of 3.5X to 22.5X. A hand tally counter (unbranded, purchased from Amazon.com) was used to make counting easier. The amount of time required to count the total *B. tabaci* eggs on each leaf disc was also recorded.

Leaf discs were photographed using the microscope set at the 0.7x minimum zoom setting and the attached 18 megapixel camera (Amscope MU1803). A 0.5x reduction adapter (Amscope FMA050) was used to increase the field of view, allowing the entire *B. tabaci* exposure area to be captured in a single photograph. The leaf images have a field of view of 39.6 mm x 29.7 mm and an image size of 4912  pixels ×  3684 pixels, resulting in a specimen resolution of 124.8 pixels per mm.

### Staining and clearing

Upon the recommendation of Sean Prager and Berenice Romero (*personal communication*), we tested a protocol previously used to stain leafhopper eggs consisting of submerging leaves in a staining solution, followed by clearing the stain from the non-egg tissues using a separate clearing solution under heat and pressure. The staining solution was originally developed by Dr. Mary C. McBryde and is made of glacial acetic acid (Fisher Chemical, Fair Lawn, NJ, USA), 200 proof ethanol (Deacon Labs, King of Prussia, PA, USA), 0.2% aqueous acid Fuchsin (Electron Microscopy Sciences, Hatfield, PA, USA), and DI water mixed at a 20:19:2:1 ratio (henceforth: McBryde’s stain) [37, 38]. The addition of 1:42nd DI water is a modification of the original method to allow use of 200 proof ethanol instead of 190 proof.

After counting and imaging the unstained leaf discs, McBryde’s stain was poured into each petri dish containing a leaf disc until the dish was about half full, about 15 ml per dish. Using a pair of tweezers, the leaf discs were carefully pressed under the stain at a slight angle so large bubbles could escape from underneath the leaf discs. The petri dishes were closed with glass lids and kept stacked in the lab fume hood at 25 °C +/- 2 °C for three days. After the three days of staining, working in a fume hood, the stain was poured out of the petri dishes, using forceps to prevent the leaf discs from coming out with the stain.

The clearing solution consists of L-lactic acid (Fujifilm Wako Chemical, Richmond, VA, USA), glycerol (Fisher Chemical, Fair Lawn, NJ, USA) and DI water mixed at a 17:20:23 ratio (henceforth: LGW) [38]. This is a slight modification of the original method to allow use of pure lactic acid instead of an 85% lactic acid aqueous solution. After the staining process, the petri dishes containing the leaf discs were filled about half full of LGW and the leaf discs were submerged under the LGW using tweezers.

The samples containing the leaf discs in LGW were then placed into steamer Insert pans (ECOZOI, Lenader, TX, USA). Then those steamer pans were placed into a preheated Instant Pot® with a small amount of water in the base [46]. After closing the lid, the Instant Pot® was set to “Pressure Cook’’ and “High Pressure” for 15 minutes. After the cook time ended, the pressure was released and samples were moved to a fume hood. LGW was then poured off and the samples were submerged in 15 ml of food grade mineral oil (Bluewater Chemgroup, Fort Wayne, IN, USA) was added to each petri dish to submerge the leaf discs. The addition of mineral oil makes it easier to view and image the samples under the microscope.

### Comparison of unstained and stained egg counts across crops

To determine if the McBryde’s staining and LGW clearing process makes *B. tabaci* egg counting more accurate and efficient, *B. tabaci* eggs were counted on *B. tabaci* exposed leaf discs before and after staining across all five crops. Plant growth, *B. tabaci* exposures, staining, and egg counts were all conducted as described in previous sections. Eggs were counted both before and after the staining and clearing process as described in the “Egg Counts and Leaf Imaging” section. Visible differences were also noted and used to assess the staining treatment.

### Analysis, statistics, and figures

All statistical analyses were conducted using R (version 4.3.2, 2023-10-31) and the packages: “dplyr”, “lme4”, “rstatix”, and “tidyr” [47–51]. An alpha of 0.05 was used as the threshold for statistical significance.

To compare the efficacy of counting McBryde’s stained *B. tabaci* eggs with counting unstained eggs, a repeated measures design was used with all the leaf discs being counted both before and after the staining treatment by two individuals. Initially, parametric statistical tests were attempted (ANOVA, regression, etc.) for this data, but it was found that the data violate normality of residual assumptions required for these tests. Two-sample Wilcoxon signed-rank tests were used to evaluate the egg counts before and after the staining treatments. The two-sample Wilcoxon signed-rank test uses ranking to compare the medians of two populations using paired samples without assuming the data are normally distributed, and is considered a non-parametric equivalent to a paired Student’s t-test. Another derived variable was calculated to examine changes in precision: “count percent difference” = (|egg count counter two - egg count counter one|) ÷ ((egg count counter two + egg count counter one) ÷ 2). Count percent difference values closer to zero represent higher between counter precision (agreement). Wilcoxon signed-rank tests were used on the paired “count percent differences” to detect significant changes in precision before and after the staining treatment.

Graphs were created using the R packages “cowplot”, “ggplot2”, “ggsignif”, “ggtext” [52–55]. Figures containing images were constructed using Affinity Designer 2 (version 2.1.1, Serif, West Bridgford, United Kingdom). To create figures, images were resized, reoriented, or arranged; no modifications were made to image color, exposure, contrast, brightness or any other relevant parameters. The ‘most representative’ before and after images for each crop were chosen for display in this publication by calculating the average change in egg count after staining, then picking the image pair closest to that average (Fig. 4). ‘Best’ and ‘worst’ example images were selected using the same criteria and are included in Figure S8.

### Staining process cost and time analysis

Costs for all equipment, supplies, and reagents required for the staining process were calculated by searching Fisher Scientific (www.fishersci.com), Millipore Sigma (www.sigmaaldrich.com), and Amazon (www.amazon.com). The website with the lowest cost for each item was used to calculate the per unit cost. Purchase quantities used for cost information were for about 100 to 200 samples (larger bulk purchases may further reduce cost). Price searches were all made on 12 Sept. 2023.

To determine the time requirement for the staining method, egg counting times were recorded for all stained and unstained egg counts. Additionally, a mock staining and clearing procedure was conducted including mixing McBryde’s stain, mixing LGW, and pouring solutions in and out of the petri plates. During the mock procedure all active time was tracked and recorded. The time required for waiting steps were not included in this analysis. This analysis of labor requirements did not include any part of the oviposition exposure experiments, as those will be the same irrespective of egg counting method.

## Results

### Comparison of unstained and stained egg counts

**Fig. 2:**
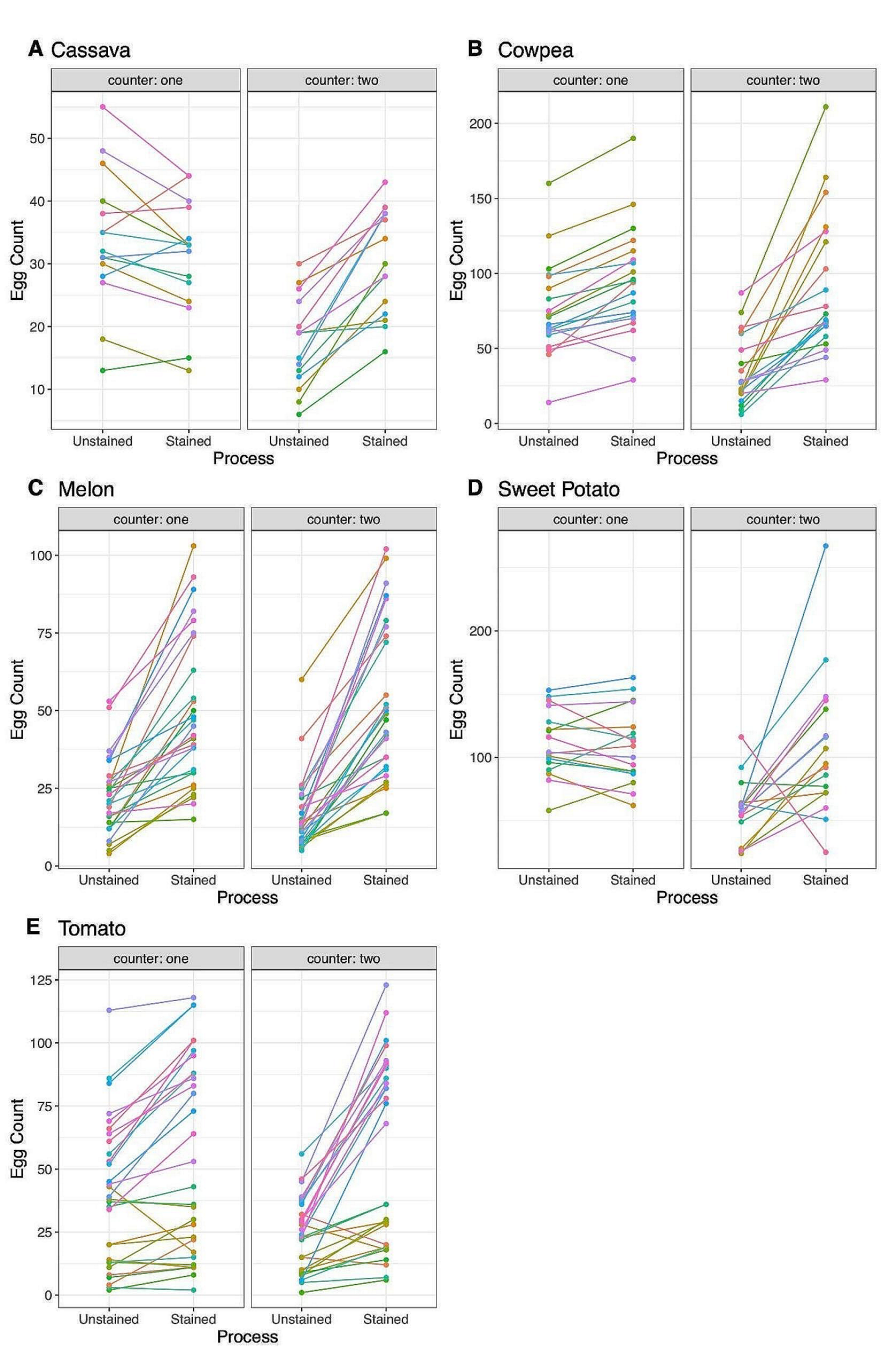
Egg counts before and after staining for each counting individual. **A**. Cassava, *n* = 15. **B**. Cowpea, *n* = 20. **C**. Melon, *n* = 28. **D**. Sweet potato, *n* = 17. **E**. Tomato, *n* = 30

We analyzed the egg count values before and after the staining process, expecting the post-staining count values to increase if staining makes eggs more visible (Fig. 2). First, we looked at the data from all experiments across all crops and used a two-sided Wilcoxon signed-rank test to perform a paired comparison of egg counts before and after staining. Egg counts were significantly higher after staining for both the more experienced counter one (*V* = 942, *p* < 0.001), and for the less experienced counter two (*V* = 178.5, *p* < 0.001). For the more experienced counter one, a significant increase in egg counts after staining was found in melons (*V* = 0.0, *p* < 0.001), tomatoes (*V* = 38.5, *p* < 0.001) and cowpeas (*V* = 10.5, *p* < 0.001), but no significant difference was found in cassava (*V* = 91.0, *p* = 0.083) or sweet potato (*V* = 91.5, *p* = 0.492) (Table S2). For counter one, egg counts increased after staining for every counted leaf disc in melons. For the less experienced counter two, a significant increase in egg counts after staining was found for cassava (*V* = 0.0, *p* < 0.001), cowpea (*V* = 0.0, *p* < 0.001), melon (*V* = 0.0, *p* < 0.001), tomato (*V* = 18.5, *p* < 0.001) and sweet potato (*V* = 19.5, *p* = 0.007) (Table S3). For counter two, egg counts increased after staining for every counted leaf disc in cassava, cowpea, and melon.

**Fig. 3:**
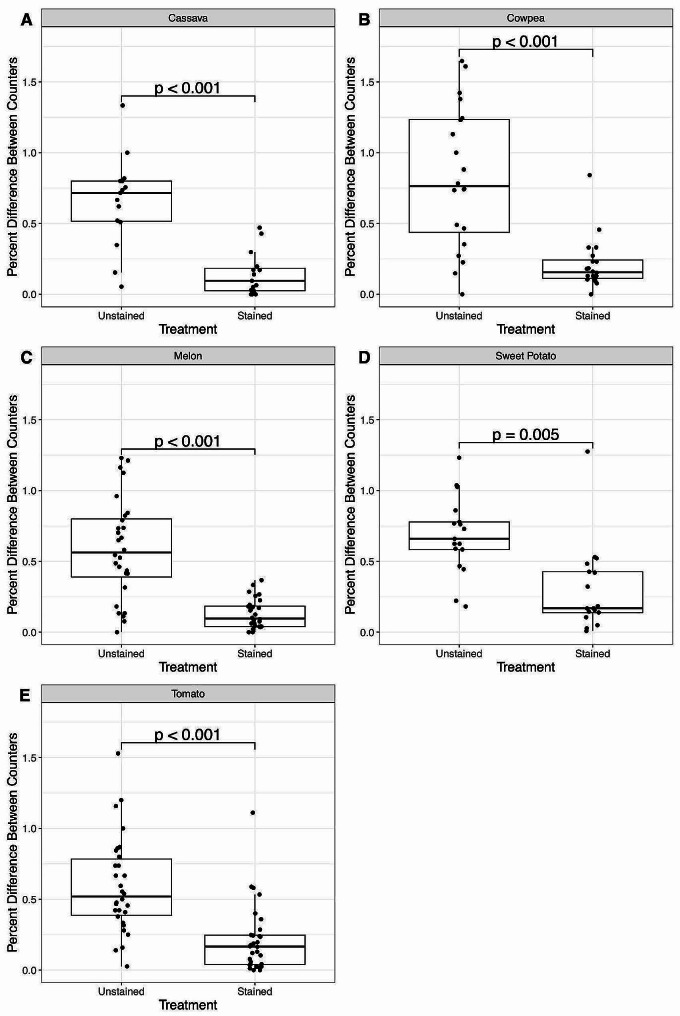
Percent difference between egg counts from each individual before and after staining. *P*-values were calculated using a Wilcoxon signed-rank test. **A**. Cassava. **B**. Cowpea. **C**. Melon. **D**. Sweet potato. **E**. Tomato

The percent difference in egg count values between the more and less experienced counters was expected to decrease if staining makes egg counting easier (representing greater agreement between the two individuals) (Fig. 3). Analysis of the percentage difference between counters across all crops using a two-sided Wilcoxon signed-rank test found a significant decrease after staining (*V* = 5731, *p* < 0.001). Analyzing each crop individually, there was a significant decrease in percent differences after staining for all crops (cassava, cowpea, melon, tomato: *p* < 0.001; sweet potato: *p* = 0.005), with over 85% of samples having lower percent differences after the staining process (Table S4).

**Fig. 4:**
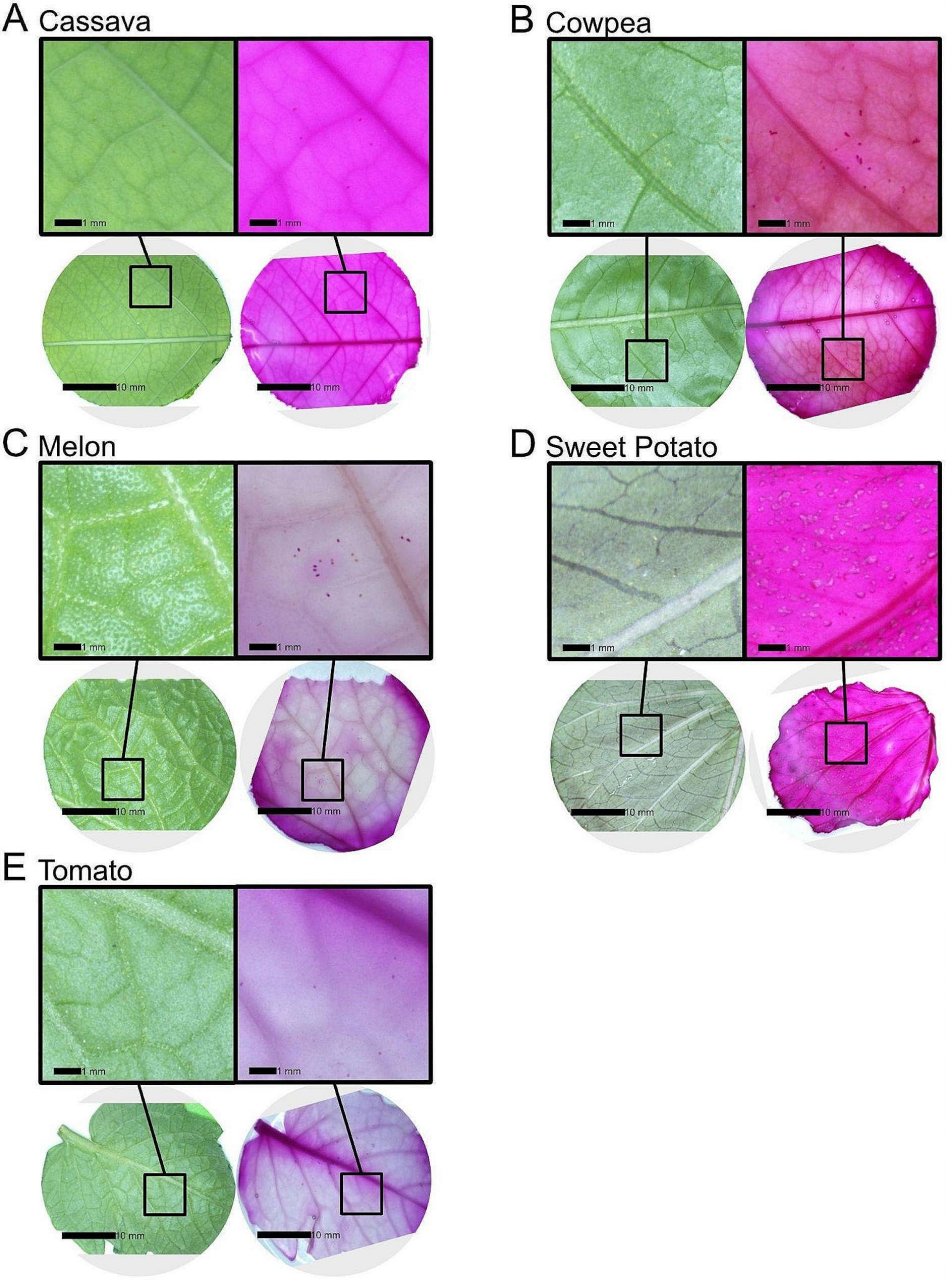
Images of excised leaf discs with *B. tabaci* eggs before (**left**) and after (**right**) treatment with McBryde’s stain and clearing solution. *B. tabaci* exposures were always on the 3rd or 4th most apical leaf with a diameter of at least 40 mm. The leaf selected for display for each crop had an egg count difference between unstained and stained that was closest to the crop average and was therefore considered representative. **A**. Cassava. **B**. Cowpea. **C**. Melon. **D**. Sweet potato. **E**. Tomato

Observations of the leaf and egg response to staining were made using the microscope images taken before and after staining. The ‘most representative’ images were selected for display in Fig. 4, and the ‘best’ and ‘worst’ stained images are provided in Figure S7. Cassava, cowpea, melon and tomato all responded well to the staining and clearing process with reasonable leaf integrity and substantial removal of green pigments. Sweet potato leaves did not respond well to the clearing component of the process and there were excessive bubbles and shriveling of the leaves (Fig. 4D and Figure S7 D). Due to the light color and lack of rough features on cassava leaves, eggs were relatively easy to see on the unstained leaves (Fig. 4A), possibly explaining the relatively small increase in count values for cassava (Tables S2 and S3).

### Costs and time of the staining method

Costs for all equipment, supplies, and reagents for this staining method, along with the labor requirements, were calculated to determine the accessibility and feasibility of this method. The total upfront equipment costs are 1577.46 USD and include the Instant Pot® and accessories, petri dishes, the microscope and the microscope camera. The microscope camera is not necessary for all phenotyping applications, potentially bringing equipment costs to just 1177.47 USD. Reagent costs include all components of the McBryde’s stain, LGW clearing solution, and mineral oil microscopy/imaging solution. Per sample reagent costs are about 1.78 USD.

The average time required to count all the eggs on an unstained leaf disc is 158 s. On average 82 s is required to count eggs on a stained leaf disc. The setup time for the staining method (including mixing of the stain and LGW and cleaning up) is about 1281 s (21.4 min). The average staining and clearing processing time for a single sample is 97 s. The total time required to obtain stained egg counts is 1281 s of setup plus 97 s of per sample processing time and 82 s of per sample egg counting time (time stained = 1282 s setup + (97 s process x n) + (82 s counting x n)). The total time to obtain unstained egg counts is 158 s per sample (time unstained = 158 s counting x n). With typical numbers of samples of around 30 to 70 per experiment, counting and process time for the stained leaf discs is only slightly longer than for unstained leaf discs (Figure S8). For example, to process and count 30 leaf discs unstained takes about 79 min, compared to about 107 min to count the same samples stained. For 70 samples, the process and count time unstained is about 183 min, compared to 221 min stained.

## Discussion

The most beneficial application of our *B. tabaci* egg staining method is in plant breeding programs, where improved precision phenotyping methods have several benefits. A key concept in plant breeding is heritability (*h*^2^) which is a measure of the proportion of a measured phenotype that is attributable to genetic variation [39]. Increasing the precision of phenotype measurements in plant breeding trials reduces measurement error, increasing *h*^2^ and therefore improving response to selection. We have reviewed ample evidence that no-choice oviposition is a valid response variable to detect host plant resistance to *B. tabaci* [14–19]. Our staining method will improve precision of the egg count measurements and therefore improve *h*^2^. Reduced measurement error is also useful because it allows detection of small differences between resistant and susceptible genotypes. Detection of these small differences can be very useful when mapping molecular markers for complex traits like host plant resistance, which may be controlled by many minor genes [56].

Our adaptation of the staining method is also low-cost and does not require specialized equipment, making the method accessible for many breeding programs. Cost might be further reduced by reusing the McBryde’s staining solution, but we did not test this for this manuscript; other studies have reused acid fuchsin based staining solutions three times [57]. We have also found the McBrydes stain to be shelf stable for long periods, with no negative effects from using staining solution mixed several months ahead. Based on our estimates of the time required to perform the final steps in the *B. tabaci* exposure assays and the leaf disc sample processing times, an individual with training and experience similar to the authors can process around 150 leaf discs using our staining method in an eight hour work day. Therefore, the biggest limit on throughput of resistance phenotyping efforts is setting up the *B. tabaci* exposure assays, which we have found to take about one working day for a single individual to perform on 60 plants. In the future, automated egg counting algorithms could be developed to reduce labor and human error.

Another application of this egg staining method is in the validation of other rapid quantification methods. Recent work by Devi et al. [58] successfully demonstrated the use of an automated *B. tabaci* egg quantification algorithm on tomatoes. ‘True’ egg count values used for algorithm development and validation in Devi et al. were based on unstained counts by humans, but our results showed that on tomato unstained egg counts are likely to be undercounts. The Devi et al. study is a valid proof of concept, especially for tomatoes, but using their method could create a larger issue when attempting to extend algorithmic counting methods to other crops, such as melon, where unstained eggs are even harder to count. We suggest future research could use McBryde’s-stained egg counts as the ‘true’ egg count values to validate automated counting methods. In these scenarios, the leaf images can be paired and aligned using the venation patterns to allow for object-level validation.

There are several aspects of this staining process that we did not test in this study, some of which may limit applications. *B. tabaci* eggs are attached to the leaf surface with a small pedicel that can uptake water [59]. There is the possibility that the staining process could interfere with this pedicel and wash eggs from the leaf surface. In our experiments on melon leaves, egg counts universally increased after staining, suggesting that the McBryde’s stain and LGW clearing process is unlikely to be washing off eggs. However, we did not directly evaluate egg retention in this study. Another limitation of our method is that it cannot account for egg viability. The staining process will necessarily kill the eggs, and we did not test if it is possible to distinguish viable from non-viable *B. tabaci* eggs after staining.

## Conclusions

We demonstrated that the McBryde’s stain and LGW clearing process can be used to visualize *B. tabaci* eggs and improve egg detection during phenotyping applications. We also showed acceptable results when clearing stained leaf tissue using an inexpensive and reliable Instant Pot®. In particular, this method is useful on melons, where counting increases were universal across samples (Fig. 2C). On this crop, improvement in contrast between the eggs and leaf surface is readily visible (Fig. 4C). The McBryde’s stain and LGW clearing process is also useful on both tomato and cowpea leaves. Our results showed less consistent improvements in egg counting on sweet potato and cassava. We suspect this is because cassava and sweet potato have smooth leaves lacking features likely to obscure *B. tabaci* eggs, and therefore the unstained egg counts are relatively accurate. We are unsure what features of the sweet potato leaves caused the shriveling response in that crop species during the staining procedure, and therefore recommend preliminary testing of the staining and clearing process on a small number of *B. tabaci* infested leaves before applying this method to a new study system. In general, our results show that this method is useful in crop species where trichomes or other aspects of the leaf anatomy make *B. tabaci* egg counting difficult.

Our staining method is effective, low cost, and applicable to breeding melons, tomatoes, and cowpeas for *B. tabaci* resistance and many other research questions that require quantification of *B. tabaci* eggs on the leaves of those species. With some adaptation and validation, we expect this method could also be extended to many other plant species where leaf traits make it difficult to count *B. tabaci* eggs. Advances in computer vision also hold promise to facilitate further improvements in speed and accuracy of *B. tabaci* egg counting and could be paired with egg staining to allow use of lower resolution microscopy and imaging equipment than would be required for automated counting of unstained eggs.

### Electronic supplementary material

Below is the link to the electronic supplementary material.

Supplementary Material 1

## Data Availability

The datasets generated and analyzed during this study, and an R Markdown document containing the code used to perform these analyses are available in a Dryad repository (DOI: doi:https://doi.org/10.5061/dryad.vmcvdnd1m).

## References

[1] Bellows TS, Perring TM, Gill RJ, Headrick DH (1994). Description of a species of *Bemisia* (Homoptera: Aleyrodidae). Ann Entomol Soc Am.

[2] Sani I, Ismail SI, Abdullah S, Jalinas J, Jamian S, Saad N. A review of the biology and control of whitefly, *Bemisia tabaci* (Hemiptera: Aleyrodidae), with special reference to biological control using entomopathogenic fungi. Insects. 2020;11. 10.3390/insects11090619 10.3390/insects11090619 PMC7564875 32927701

[3] Greathead AB. Host plants. In: Cock MJW, editor. *Bemisia tabaci* – a literature survey on the cotton whitefly with an annotated bibliography. C.A.B International Institute of Biological Control; 1986. pp. 17–25.

[4] Padilha G, Pozebon H, Patias LS, Ferreira DR, Castilhos LB, Forgiarini SE (2021). Damage assessment of *Bemisia tabaci* and economic injury level on soybean. Crop Prot.

[5] Li Y, Mbata GN, Punnuri S, Simmons AM, Shapiro-Ilan DI. *Bemisia tabaci* on vegetables in the Southern United States: incidence, impact, and management. Insects. 2021;12. 10.3390/insects12030198 10.3390/insects12030198 PMC7996905 33652635

[6] Lourenção AL, Alves AC, Melo AMT, Valle GE (2011). Development of leaf silvering in squash cultivars infested by silverleaf whitefly. Hortic Bras.

[7] Chen J, McAuslane HJ, Carle RB, Webb SE (2004). Impact of *Bemisia Argentifolii* (Homoptera: Auchenorrhyncha: Aleyrodidae) infestation and squash silverleaf disorder on zucchini yield and quality. J Econ Entomol.

[8] Nombela G, Muñiz M, Stansly PA, Naranjo SE (2010). Host plant resistance for the management of *Bemisia tabaci*: a multi-crop survey with emphasis on tomato. *Bemisia*: bionomics and management of a global pest.

[9] Simmons AM, Kousik CS, Levi A (2010). Combining reflective mulch and host plant resistance for sweetpotato whitefly (Hemiptera: Aleyrodidae) management in watermelon. Crop Prot.

[10] Horowitz AR, Antignus Y, Gerling D. Management of *Bemisia tabaci* whiteflies. In: Thompson WMO, editor. The whitefly, *Bemisia tabaci* (Homoptera: Aleyrodidae) interaction with geminivirus-infected host plants: *Bemisia tabaci*, host plants and geminiviruses. Dordrecht: Springer Netherlands; 2011. pp. 293–322.

[11] Teetes GL. Plant resistance to insects: a fundamental component of IPM. Radcliffe’s IPM world textbook. St. Paul, MN: University of Minnesota; 2006 [cited 2023 Oct 30]. https://ipmworld.umn.edu/teetes

[12] Smith CM, Smith CM (2005). Plant resistance to arthropods: Molecular and conventional approaches.

[13] Bellotti AC, Arias B (2001). Host plant resistance to whiteflies with emphasis on cassava as a case study. Crop Prot.

[14] Nombela G, Beitia F, Muniz M (2001). A differential interaction study of *Bemisia tabaci* Q-biotype on commercial tomato varieties with or without the Mi resistance gene, and comparative host responses with the B-biotype. Entomol Exp Appl.

[15] Momotaz A, Scott JW, Schuster DJ. Searching for silverleaf whitefly and begomovirus resistance genes from *Lycopersicon hirsutum* accession LA1777. ISHS Acta Horticulturae. 2005 [cited 2022 Nov 6];695. https://www.actahort.org/books/695/695_51.htm

[16] Novaes NS, Lourenção AL, Bentivenha JPF, Baldin ELL, Melo AMT (2020). Characterization and potential mechanisms of resistance of cucumber genotypes to *Bemisia tabaci* (Hemiptera: Aleyrodidae). Phytoparasitica.

[17] Rodríguez-Álvarez CI, Muñiz M, Nombela G (2017). Effect of plant development (age and size) on the Mi-1-mediated resistance of tomato to whitefly *Bemisia tabaci*. Bull Entomol Res.

[18] Silva JPG, Baldin ELL, de Souza ES, Lourenção AL (2012). Assesing *Bemisia tabaci* (Genn.) Biotype B resistance in soybean genotypes: antixenosis and antibiosis. Chil J Agricultural Res.

[19] Coelho SAMP, Lourenção AL, de Melo AMT, Schammass EA (2009). Resistência De Meloeiro a *Bemisia tabaci* biótipo B. Bragantia.

[20] Carolina Farias e Silva M, de Sousa Rodrigues A, Henrique Ferreira Rodrigues R, Ettore Pavan B, Barboza Silva L (2023). Performance of *Bemisia tabaci* MEAM1 on soybean and resistance traits of cultivars. J Asia Pac Entomol.

[21] Sauvion N, Mauriello V, Renard B, Boissot N (2005). Impact of melon accessions resistant to aphids on the demographic potential of silverleaf whitefly. J Econ Entomol.

[22] Cardoza YJ, McAuslane HJ, Webb SE (1999). Mechanisms of resistance to whitefly-induced squash silverleaf disorder in zucchini. J Econ Entomol.

[23] Soria C, López-Sesé AI, Gómez-Guillamón ML (1999). Resistance of *Cucumis melo* against *Bemisia tabaci* (Homoptera: Aleyrodidae). Environ Entomol.

[24] Cruz PL, Baldin ELL, de Jesus P, de Castro M (2014). Characterization of antibiosis to the silverleaf whitefly *Bemisia tabaci* biotype B (Hemiptera: Aleyrodidae) in cowpea entries. J Pest Sci.

[25] Jeevanandham N, Marimuthu M, Natesan S, Gandhi K, Appachi S (2018). Plant resistance in chillies *Capsicum* spp. against whitefly, *Bemisia tabaci* under field and greenhouse condition. J Entomol Zool Stud.

[26] Palomares-Rius FJ, López-Sesé AI, Gómez-Guillamón ML (2010). Preliminary study of resistance against *Bemisia tabaci* Genn. In TGR-1551 melon genotype. Acta Hortic.

[27] Boissot N, Thomas S, Sauvion N, Marchal C, Pavis C, Dogimont C (2010). Mapping and validation of QTLs for resistance to aphids and whiteflies in melon. Theor Appl Genet.

[28] Abubakar M, Koul B, Chandrashekar K, Raut A, Yadav D (2022). Whitefly (*Bemisia tabaci*) management (WFM) strategies for sustainable agriculture: a review. Collect FAO Agric.

[29] Luckew A, Meru G, Wang Y-Y, Mwatuwa R, Paret M, Carvalho R (2022). Field evaluation of *Cucurbita* germplasm for resistance to whiteflies and whitefly-transmitted viruses. HortScience.

[30] Riley D, Batal D, Wolff D (2001). Resistance in glabrous-type *Cucumis melo* L. to whiteflies (Homoptera: Aleyrodidae). J Entomol Sci.

[31] McCreight JD, Wintermantel WM, Natwick ET. Host plant resistance in melon to sweetpotato whitefly in California and Arizona. Acta Hortic. 2017:237–44.

[32] Boissot N, Lafortune D, Pavis C, Sauvion N (2003). Field resistance to *Bemisia tabaci* in *Cucumis melo*. HortScience.

[33] Sippell DW, Bindra OS, Khalifa H (1987). Resistance to whitefly (*Bemisia tabaci*) in cotton (*Gossypium hirsutum*) in the Sudan. Crop Prot.

[34] Cuthbertson A. *Bemisia tabaci* (MEAM1) (silverleaf whitefly). CABI Compendium. 2015 [cited 2023 Feb 8]. https://www.cabidigitallibrary.org/doi/10.1079/cabicompendium.8925

[35] Coe MT, Evans KM, Gasic K, Main D (2020). Plant breeding capacity in U.S. public institutions. Crop Sci.

[36] De Barro PJ, Liu S-S, Boykin LM, Dinsdale AB (2011). *Bemisia tabaci*: a statement of species status. Annu Rev Entomol.

[37] McBryde MC (1936). A method of demonstrating rust hyphae and haustoria in unsectioned leaf tissue. Am J Bot.

[38] Backus EA, Hunter WB, Arne CN (1988). Technique for staining leafhopper (Homoptera: Cicadellidae) salivary sheaths and eggs within unsectioned plant tissue. J Econ Entomol.

[39] Bernardo R. Essentials of plant breeding. Stemma; 2014.

[40] La Bonte DR, Clark CA, Smith TP, Villordon AQ, Scott Stoddard C (2015). Bellevue Sweetpotato HortScience.

[41] Matkin OA, Chandler PA. The U.C-type soil mixes. In: Baker KF, editor. The U C system for producing healthy container-grown plants. University of California Division of Agricultural Sciences; 1957. pp. 68–85.

[42] MacGillivray ME, Anderson GB (1957). Three useful insect cages. Can Entomol.

[43] Muñiz M, Nombela G. *Bemisia tabaci*: A new clip-cage for biological studies. 2001 [cited 2023 Sep 1]; https://digital.csic.es/handle/10261/11859

[44] Cass BN, Himler AG, Bondy EC, Bergen JE, Fung SK, Kelly SE (2016). Conditional fitness benefits of the *Rickettsia* bacterial symbiont in an insect pest. Oecologia.

[45] Veenstra KH, Byrne DN (1998). Effects of starvation and oviposition activity on the reproductive physiology of the sweet potato whitefly, *Bemisia tabaci*. Physiol Entomol.

[46] Swenson VA, Stacy AD, Gaylor MO, Ushijima B, Philmus B, Cozy LM (2018). Assessment and verification of commercially available pressure cookers for laboratory sterilization. PLoS ONE.

[47] R Core Team. R: a language and environment for statistical computing. Vienna, Austria: R Foundation for Statistical Computing. 2023. https://www.R-project.org/

[48] Wickham H, François R, Henry L, Müller K, Vaughan D. dplyr: a grammar of data manipulation. 2023. https://CRAN.R-project.org/package=dplyr

[49] Wickham H, Vaughan D, Girlich M. tidyr: tidy messy data. 2023. https://CRAN.R-project.org/package=tidyr

[50] Bates D, Mächler M, Bolker B, Walker S. Fitting linear mixed-effects models using lme4. J Stat Softw. 2015. p. 1–48.

[51] Kassambara A, rstatix. Pipe-friendly framework for basic statistical tests. 2023. https://CRAN.R-project.org/package=rstatix

[52] Wickham H. ggplot2: elegant graphics for data analysis. Springer-Verlag New York; 2016. https://ggplot2.tidyverse.org

[53] Constantin A-E, Patil I. ggsignif: R package for displaying significance brackets for ’ggplot2’. PsyArxiv. 2021. https://psyarxiv.com/7awm6

[54] Wilke CO, Wiernik BM. ggtext: improved text rendering support for ggplot2. 2022. https://CRAN.R-project.org/package=ggtext

[55] Wilke CO. cowplot: streamlined plot theme and plot annotations for ggplot2. 2020. https://CRAN.R-project.org/package=cowplot

[56] Broman KW (2009). A guide to QTL mapping with R/qtl.

[57] Moorthy KPN, Alexander MP, Tewari GC (1988). Versatile method for staining insect eggs and larvae in leaves. J Econ Entomol.

[58] Devi MG, Rustia DJA, Braat L, Swinkels K, Espinosa FF, van Marrewijk BM (2023). Eggsplorer: a rapid plant-insect resistance determination tool using an automated whitefly egg quantification algorithm. Plant Methods.

[59] Buckner JS, Freeman TP, Ruud RL, Chu C-C, Henneberry TJ (2002). Characterization and functions of the whitefly egg pedicel. Arch Insect Biochem Physiol.

