# Supplementary material for: Evaluation of a low-cost staining method for improved visualization of sweet potato whitefly (Bemisia tabaci) eggs on multiple crop plant species
Source: Plant Methods. 2024 May 23;20:75. doi: 10.1186/s13007-024-01209-z (PMC11112839; doi:10.1186/s13007-024-01209-z)
Supplement: Supplementary file 1 — Supplementary Material 1 [file 13007_2024_1209_MOESM1_ESM.docx]

Supplemental Figures and Tables:
Evaluation of a Low-Cost Staining Method for Improved Visualization of Sweet Potato Whitefly (*Bemisia tabaci*) Eggs on Multiple Crop Plant Species

Benjamin van Raalte^1^, Kristal Watrous^2^, Miguel Lujan^1^, Ricky Le^1^, Penglin Sun^1^, Benjamin Ellis^3^, and Kerry Mauck^1,4^

1. University of California, Riverside, Department of Entomology. 165 Entomology Bldg., Citrus Drive, Riverside, CA 92521, USA

2. University of California, Irvine, Department of Ecology and Evolutionary Biology. 321 Steinhaus Hall, Irvine, CA 92697, USA

3. University of California, Riverside, Department of Statistics. 900 University Ave., Olmsted Hall 1337, Riverside, CA 92521, USA

4. Author for correspondence: kerry.mauck@ucr.edu

#

# Supplemental Methods

## Constructing Clip Cages

Cages were constructed out of two Corning™ Falcon™ 50 ml conical centrifuge tubes (Corning Incorporated, NY, NY, USA) (henceforth “Falcon tube”) cut off at the 35 ml mark and then glued together with a fine 210 micron plastic screen (U.S. mesh size 70) separating the two sides (Figure S1 A). The internal volume on each side of the mesh is about 45 ml. To make the leaf clips, 4.76 mm thick polyurethane foam (76 Flex-Foam™, Pellon, Clearwater, FL, USA) (henceforth “Flex-Foam™”) was glued onto the top of one of the Falcon tube caps, and a round 24 mm hole was cut into the cap and Flex-Foam™ using a cylindrical metal punch heated to red hot with an acetylene torch, extra Flex-Foam™ was trimmed around the edges of the cap (Figure S1 B). This hole with a 24 mm diameter exposes a 452 mm^2^ area of the leaf surface to whiteflies contained in the cage. A round section of flat plastic from the lid of a food storage container was cut into a disc using a 35 mm hole saw, Flex-Foam™ was glued to the disc and trimmed to the same size. The foam-covered cap and disc were then attached together using an aluminum hair sectioning clip (Goody Products, Atlanta, GA, USA) with a bend in the clip to create a piece that can be closed onto a leaf (Figure S1 C). The other Falcon tube cap was retained unaltered for enclosing whiteflies during transport and while attaching leaf clips.

The key difference between these clip cages and other versions is that they are symmetrical and threaded on both sides. This design allows them to easily be filled using an inline aspiration device consisting of two Falcon tube caps with holes drilled in the center and teflon tubes affixed into the holes (Figure S1 D). The symmetrical cages are also easier to make in large quantities because there are fewer unique parts.

## Comparison of the autoclave to the Instant Pot^®^ for melon leaves

Use of an Instant Pot® brand electric pressure cooker (eight quart Instant Pot® Duo® 7-in-1, Instant Brands, Downers Grove, IL, USA) for clearing the leaf discs was compared to the original Backus et al. [[1]](https://paperpile.com/c/ozT3A8/JiJMe/?noauthor=1) method which utilizes an autoclave. Use of an Instant Pot® has previously been demonstrated as a low-cost autoclave substitute for laboratory sterilization applications [[2]](https://paperpile.com/c/ozT3A8/wmahO). For these comparisons, 16 melon plants (variety ‘Gold Crown’) were grown in the same manner as described in the “Plant Materials” section and exposed to whiteflies from 41 to 45 days after planting. Composition of the McBryde’s stain and LGW is as described in the “Staining and Clearing Solutions” section. After the *B. tabaci* exposures and staining, eight of the melon leaf discs were cleared using the autoclave and the other eight using the Instant Pot^®^.

For the samples receiving the autoclave treatment, petri dishes containing the leaf discs and LGW were covered with glass lids, placed on a rigid plastic tray, and that tray was placed in an autoclave (Amsco® Eagle® SV-3053, Steris, Mentor, OH, USA). The autoclave was then run on a liquid sterilization cycle which held the samples at 120 to 125 kilopascals of pressure and around 121 °C for 15 minutes. At the end of the autoclave cycle, the door was opened slightly to allow the samples to begin to cool. Once the dishes had cooled enough to handle, the tray was moved to a fume hood. LGW was then poured off the leaf discs using tweezers to keep the leaf discs in the dishes, and about 15 ml of food grade mineral oil (Bluewater Chemgroup, Fort Wayne, IN, USA) was added to each petri dish to submerge the leaf discs. The addition of mineral oil makes it easier to view and image the samples under the microscope.

The petri dishes containing the leaf disc samples receiving the Instant Pot® treatment were submerged in LGW and placed in Stackable 3 Tier Steamer Insert Pans (ECOZOI, Lenader, TX, USA). Then those steamer pans were placed into a preheated Instant Pot® with a small amount of water in the base. After closing the lid, the Instant Pot® was set to “Pressure Cook'' and “High Pressure” for 15 minutes, with “Keep Warm'' turned off. After the cook time ended, the pressure was released and samples were moved to a fume hood. LGW was then poured off and the samples were submerged in food grade mineral oil in the same manner as described for the autoclave treatment group.

After staining, the eggs appear as dark purple to black dots against a mostly clear or light purple leaf background. Stained counts were performed on both the autoclave and instant pot treatment using the same microscope setup and method as described in the “Egg Counts and Leaf Imaging” section.

## Testing surfactants to reduce bubbles on stained leaf discs

In some crops, particularly those with many leaf trichomes, we observed the formation of small bubbles on the leaf surface during the clearing process. Because of the potential for these bubbles to obscure *B. tabaci* eggs, we tested the addition of surfactants to the clearing solution or the mineral oil used during egg counting. Tween® 20 (Polysorbate 20, Fisher Chemical, Fair Lawn, NJ, USA) is soluble in water and was tested on tomato leaf discs as an addition to the LGW clearing solution at a concentration of 0.1% (v/v). Span 80 (Sorbitan Monooleate, TCI America, Portland, OR, USA) is soluble in oil and was tested as an addition to the mineral oil used during egg counting. Mixing Span 80 into the mineral oil was tested on the melon at a concentration of 0.1% (v/v) or 0.5% (v/v), and on sweet potato and cowpea at a concentration of 0.5% (v/v). When mixing the surfactants into either the LGW or the mineral oil, a stir bar was used at 200 RPM for one minute to ensure the viscous surfactants were fully dissolved into the solutions. Half the leaf disc samples were then selected at random to receive one of the surfactant treatments (Table S1). Eggs were counted both before and after staining as described in the “Egg Counts and Leaf Imaging” section. For several of these surfactant experiments an additional variable was recorded during egg counting, the number of bubbles as large or larger than a *B. tabaci* egg present on the leaf surface.

## Statistical analysis of autoclave to Instant Pot® comparisons

To compare clearing using the Instant Pot® to clearing using the Autoclave, and to assess the effect of the surfactant treatments, two response variables were used: (1) The difference between egg counts before and after staining (*stained egg count* - *unstained egg count* = *egg difference*) for each counting individual, with higher values expected if the method is effectively improving the visibility of *B. tabaci* eggs. (2) The number of bubbles on the leaf surface larger than a *B. tabaci* egg, which was counted only once by the more experienced counter one and is expected to be lower on the treatment groups where staining and clearing worked better. Initially, parametric statistical tests were attempted (ANOVA, regression, etc.), but it was found that the data violates normality of residual assumptions required for these tests. Exact two-sample Kolmogorov-Smirnov (KS) tests were used to compare the distributions of these responses for each treatment group. KS tests allow us to determine the likelihood that these data sets came from the same distribution without making assumptions about the overall type of distribution.

# Supplemental Results

## Comparing the autoclave to the Instant Pot®

To determine the best clearing method for future experiments, we did a small experiment comparing the differences between stained and unstained egg counts on melon leaf discs when clearing using the original autoclave method described in Backus et al. [[1]](https://paperpile.com/c/ozT3A8/JiJMe/?noauthor=1) to clearing using an Instant Pot®. Using a KS test we found that the distribution of the differences in egg counts from the Instant Pot® method was not significantly different to the distribution of differences from the autoclave method for either counter one (*D* = 0.50, *p* = 0.283) or counter two (*D* = 0.38, *p* = 0.622) (Figure S2 A). The mean number of bubbles larger than a *B. tabaci* egg on Instant Pot® cleared leaf discs (*M* = 31.8, *Mdn* = 25, *N* = 8) was substantially higher than on autoclave cleared leaf discs (*M* = 2.5, *Mdn* = 2, *N* = 8), and distribution of the number of bubbles for the Instant Pot® method was significantly different from the distribution for the autoclave method (*D* = 1.0, *p* < 0.001) (Figure S2 B). These results indicate that the formation of bubbles occurs more frequently when clearing using the Instant Pot® compared to the autoclave. Visual observation of the leaf surfaces showed that clearing was more complete and consistent using the autoclave (Figure S3). These experiments were only conducted on melon leaves and had low sample sizes so they may not be generalizable across all crops, but overall the autoclave demonstrates slightly better clearing characteristics than the Instant Pot®. However, because the Instant Pot® yields acceptable results at a much lower cost we used the Instant Pot® clearing method in all further experiments.

## Testing surfactants to reduce bubbles on the leaf discs

Tween® 20 is a water soluble surfactant and was tested at a concentration of 0.1% (v/v) in the LGW solution in an attempt to eliminate the bubbles on the leaf surface and improve clearing performance. Tomato leaf discs were used for this experiment. Using a KS test we found no significant difference in the distributions of the differences between stained and unstained egg counts on tomato leaf discs when clearing with 0.1% (v/v) Tween® 20 compared to without surfactant for either counter one (*D* = 0.21, *p* = 0.933) or counter two (*D* = 0.48, *p* = 0.252) (Figure S4 A). The average number of bubbles larger than a *B. tabaci* egg on the tomato leaf discs cleared with 0.1% (v/v) Tween® 20 (*M* = 89, *Mdn* = 72, *N* = 8) was lower than on the tomato leaf discs clear without surfactant (*M* = 177, *Mdn* = 165, *N* = 8) (Figure S4 B). However, the distributions of the number of bubbles were not significantly different (*D* = 0.50, *p* = 0.283).

Span 80 is an oil soluble surfactant and was tested on melons, sweet potatoes and cowpeas as an addition to the mineral oil used during egg counting with the goal of improving egg visibility and reducing bubbles. Using a KS test the differences between stained and unstained egg counts for melon leaf discs were not significantly different with 0.1% (v/v) Span 80 compared to without surfactant for either counter one (*D* = 0.36, *p* = 0.649) or counter two (*D* = 0.24, *p* = 0.962) (Figure S5 A). The average number of bubbles on the melon leaf discs with 0.1% (v/v) Span 80 (*M* = 6.25, *Mdn* = 6, *N* = 8) was lower than on the melon leaf discs without surfactant (*M* = 10.7, *Mdn* = 10, *N* = 7), but those distributions were not significantly different (*D* = 0.32, *p* = 0.681) (Figure S5 B). The surfactant group of melon leaf discs were resubmerged in mineral oil with 0.5% (v/v) Span 80 and the bubbles in both groups were counted again. The average number of bubbles on the melon leaf discs with 0.5% (v/v) Span 80 (*M* = 2.62, *Mdn* = 1, *N* = 8) was lower than on the melon leaf discs without surfactant (*M* = 6.14, *Mdn* = 3, *N* = 7), but those distributions were not significantly different (*D* = 0.34, *p* = 0.572).

The addition of 0.5% (v/v) Span 80 to the mineral oil was also tested on cowpea and sweet potato. Bubbles were not counted in this experiment so only egg count differences were compared for these experiments. Using a KS test, we found no evidence that the distributions of differences between stained and unstained egg counts are different on cowpea leaves with 0.5% (v/v) Span 80 compared to without surfactant for either counter one (*D* = 0.29, *p* = 0.809) or counter two (*D* = 0.29, *p* = 0.847) (Figure S6 A). We had similar results on sweet potato leaf discs showing no significant difference in distributions of count differences with 0.5% (v/v) Span 80 compared to without surfactant for counter one (D = 0.38, p = 0.464) and counter two (D = 0.44, p = 0.242) (Figure S6 B).

It is important to note that while we decided the results of these surfactant experiments were sufficient to proceed without surfactant additions in future experiments, the sample sizes were small so our statistical tests were underpowered to definitively rule out benefits of adding surfactants during the clearing and counting process.

# Supplemental Tables

| **Table S1**: Summary of all *B. tabaci* oviposition trials performed for this project. | | | | | | | | | | | |
| --- | --- | --- | --- | --- | --- | --- | --- | --- | --- | --- | --- |
| **Trial Name** | **Plant Species** | **Plant Variety (ies)** | **Experiment(s)** | **Surfactant Experiment** | **Exposure Start Date** | **Plant age exposure start** | **Plant age exposure end** | **Total Days of Exposure** | **Number of Plant Entries** | **Number of Clip Cages Per Entry** | **Notes** |
| Instant Pot 1 | Cowpea | uk | Preliminary | none | 5 June 2023 | uk | uk | uk | 5 | 1 |  |
| Instant Pot 1 | Melon | uk | Preliminary | none | 5 June 2023 | uk | uk | uk | 5 | 1 |  |
| Instant Pot 2 | Melon | “Gold Crown” | Instant Pot vs. Autoclave | none | 26 June 2023 | 41 | 45 | 4 | 16 | 1 |  |
| Cassava Rep 1 | Cassava | unknown | Stain:unstain | none | 15 August 2023 | 60 | 63 | 3 | 8 | 2 | One cage lost - broken leaf |
| Cowpea FS Rep 1 | Cowpea | “Mississippi Silver” | Surfactant;  Stain:unstain | 0.5% Span 80 in mineral oil | 7 August 2023 | 27 | 31 | 4 | 15 | 1 |  |
| Tomato Rep 2 pt 1 | Tomato | “Moneymaker” or “Motelle” | Surfactant;  Stain:unstain | 0.1% Tween 20 in LGW | 18 July 2023 | 63 | 66 | 3 | 15 | 1 |  |
| Tomato Rep 2 pt 2 | Tomato | “Moneymaker” or “Motelle” | Stain:unstain | none | 24 August 2023 | 97 | 101 | 4 | 13 | 1-2 |  |
| Melon TGR Rep 4 pt2 | Melon | “Top Mark” or “TGR1551” | Surfactant;  Stain:unstain | 0.1% Span 80 and 0.5% in mineral oil | 25 July 2023 | 59 | 63 | 4 | 15 | 1 |  |
| Sweet Potato Rep 1 | Sweet Potato | “Beauregard” and “Bellevue” | Surfactant;  Stain:unstain | 0.5% Span 80 in mineral oil | 31 July 2023 | 48 (from start of cutting rooting) | 52 (from start of cutting rooting) | 4 | 17 | 1 |  |

**Table S2**. Analyses of egg counts by the more experienced counter before and after staining for each crop.

| Crop Species | N  Samples | Unstained Median | Stained Median | Unstained Median Absolute  Deviation | Stained Median Absolute  Deviation | Samples Where Counts Increased (%) | V Statistic | p-value |
| --- | --- | --- | --- | --- | --- | --- | --- | --- |
| Cassava | 15 | 32 | 33 | 7.41 | 8.90 | 33.33 | 91.0 | 0.0830 |
| Cowpea | 20 | 68.5 | 94.5 | 23.72 | 31.88 | 95.00 | 10.5 | 0.0004 |
| Melon | 28 | 22 | 43.5 | 11.12 | 22.98 | 100 | 0.0 | <0.0001 |
| Sweet Potato | 17 | 104 | 109 | 25.2 | 29.65 | 47.05 | 91.5 | 0.4920 |
| Tomato | 30 | 38.5 | 48 | 37.06 | 54.11 | 80.00 | 38.5 | <0.0001 |

**Table S3**. Analyses of egg counts by the less experienced counter before and after staining for each crop.

| Crop Species | N  Samples | Unstained Median | Stained Median | Unstained Median Absolute  Deviation | Stained Median Absolute  Deviation | Samples Where Counts Increased (%) | V Statistic | p-value |
| --- | --- | --- | --- | --- | --- | --- | --- | --- |
| Cassava | 15 | 19 | 30 | 8.90 | 11.86 | 100 | 0.0 | 0.0007 |
| Cowpea | 20 | 27.5 | 71 | 18.53 | 29.65 | 100 | 0.0 | <0.0001 |
| Melon | 28 | 13 | 48 | 8.9 | 29.65 | 100 | 0.0 | <0.0001 |
| Sweet Potato | 17 | 57 | 95 | 10.38 | 34.10 | 82.35 | 19.5 | 0.0075 |
| Tomato | 30 | 23.5 | 52 | 20.02 | 49.67 | 90.00 | 18.5 | <0.0001 |

# Supplemental Figures


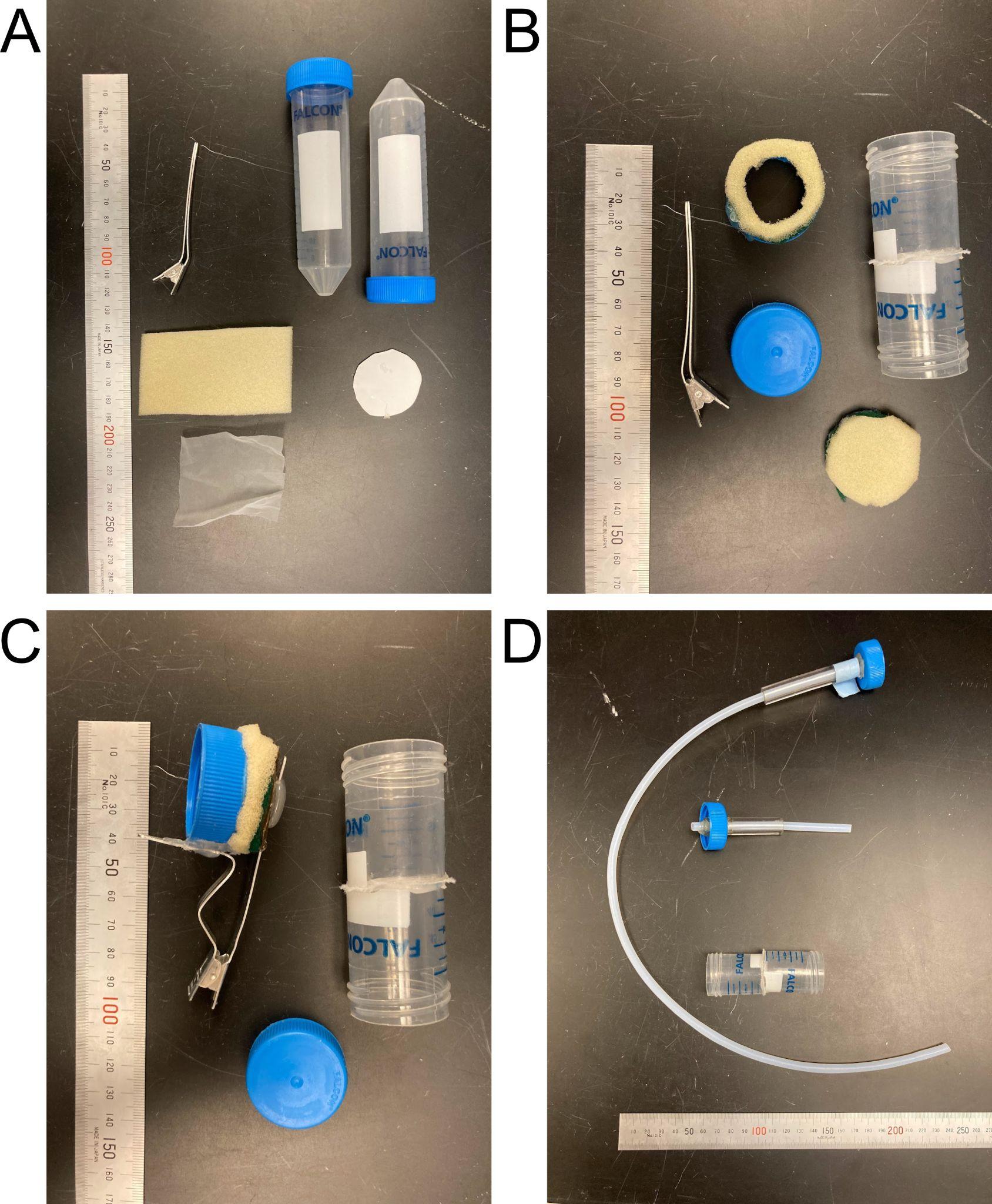


**Figure S1**. design of clip cage devices. Scales in mm. **A**. Clip cage components before cutting and assembly. **B**. Partially assembled clip cage. **C**. Fully assembled clip cage with leaf clip and cage components detached from each other. **D**. Inline aspiration attachments for filling cages with whiteflies, with a cage in the center.


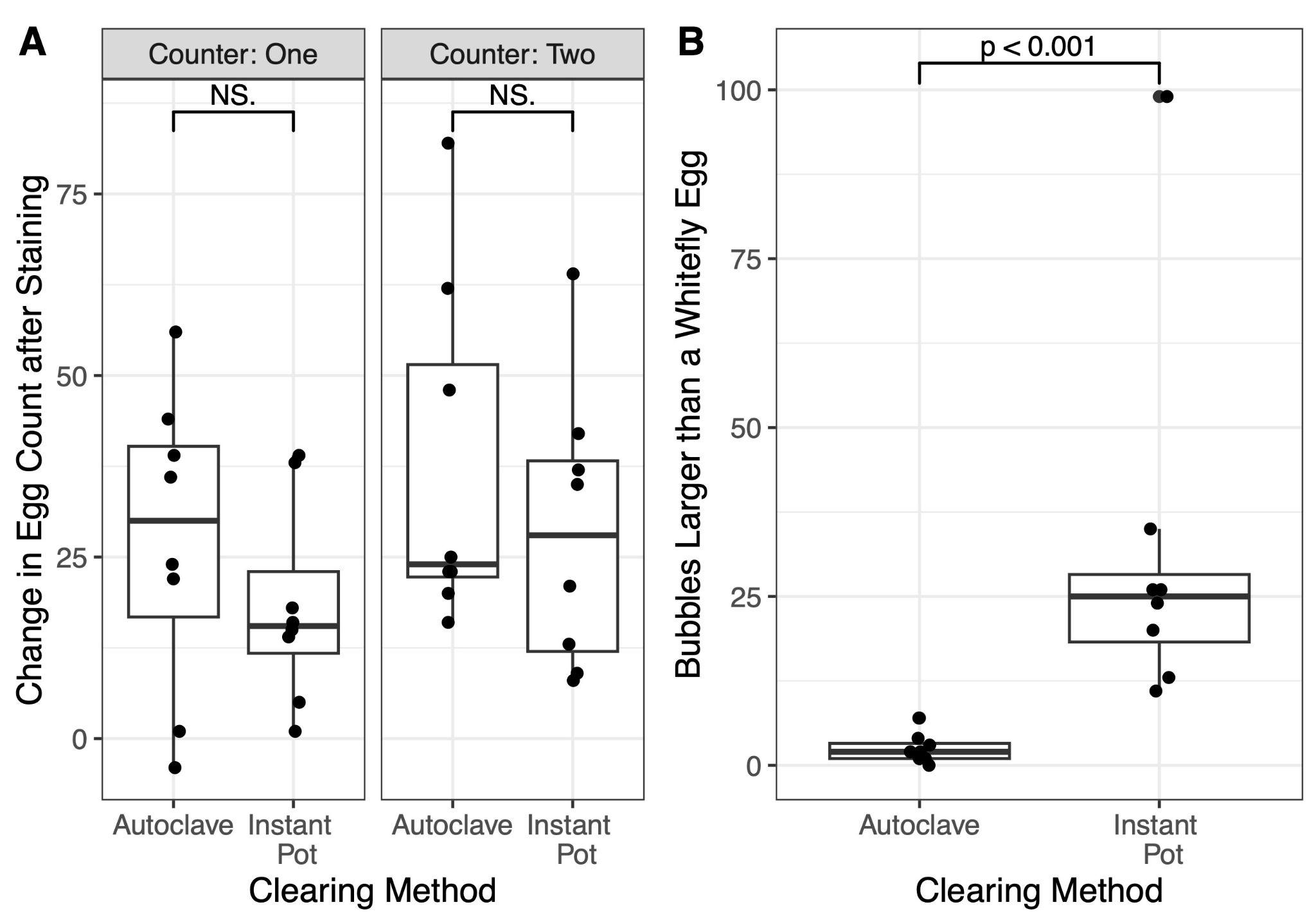


**Figure S2**. Melon leaf disc response to clearing with the autoclave and the Instant Pot. **A**. Change in egg count after the staining process for each counting individual. **B**. Number of bubbles on stained leaf discs.

**
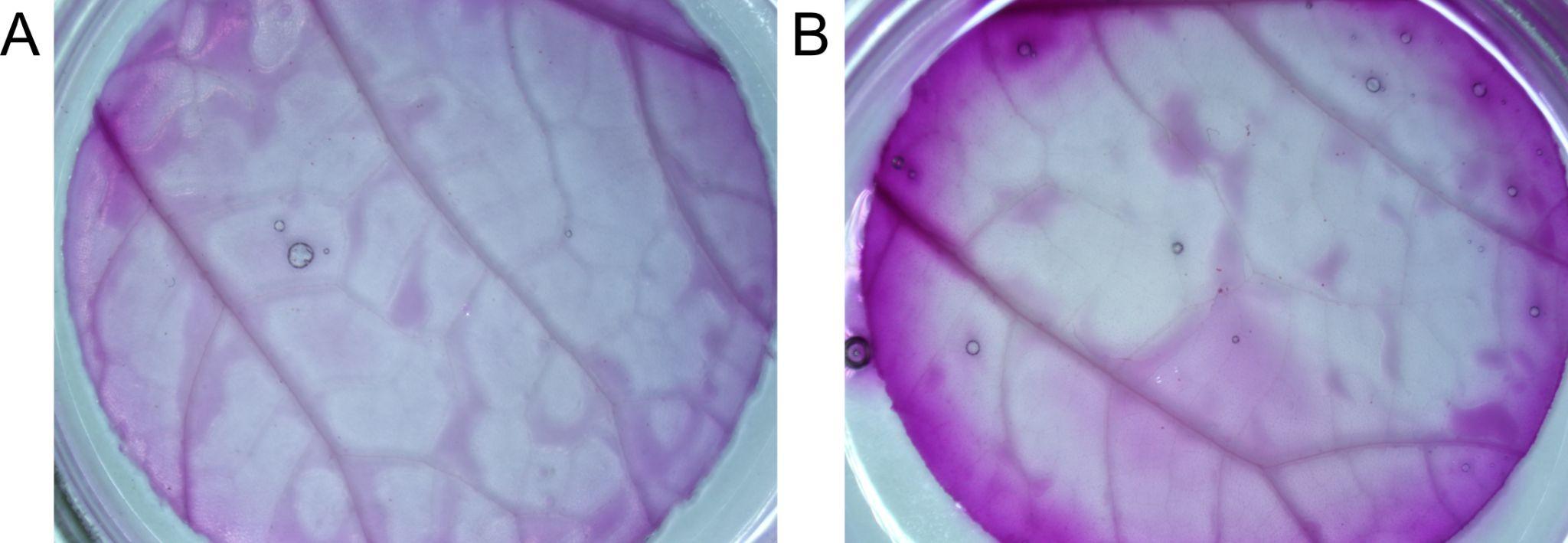
**

**Figure S3**: Images from an Instant Pot® to autoclave comparison experiment conducted on melon leaves. Displayed images selected randomly to give a general idea of the results. **A**: autoclave. **B**: Instant Pot®.


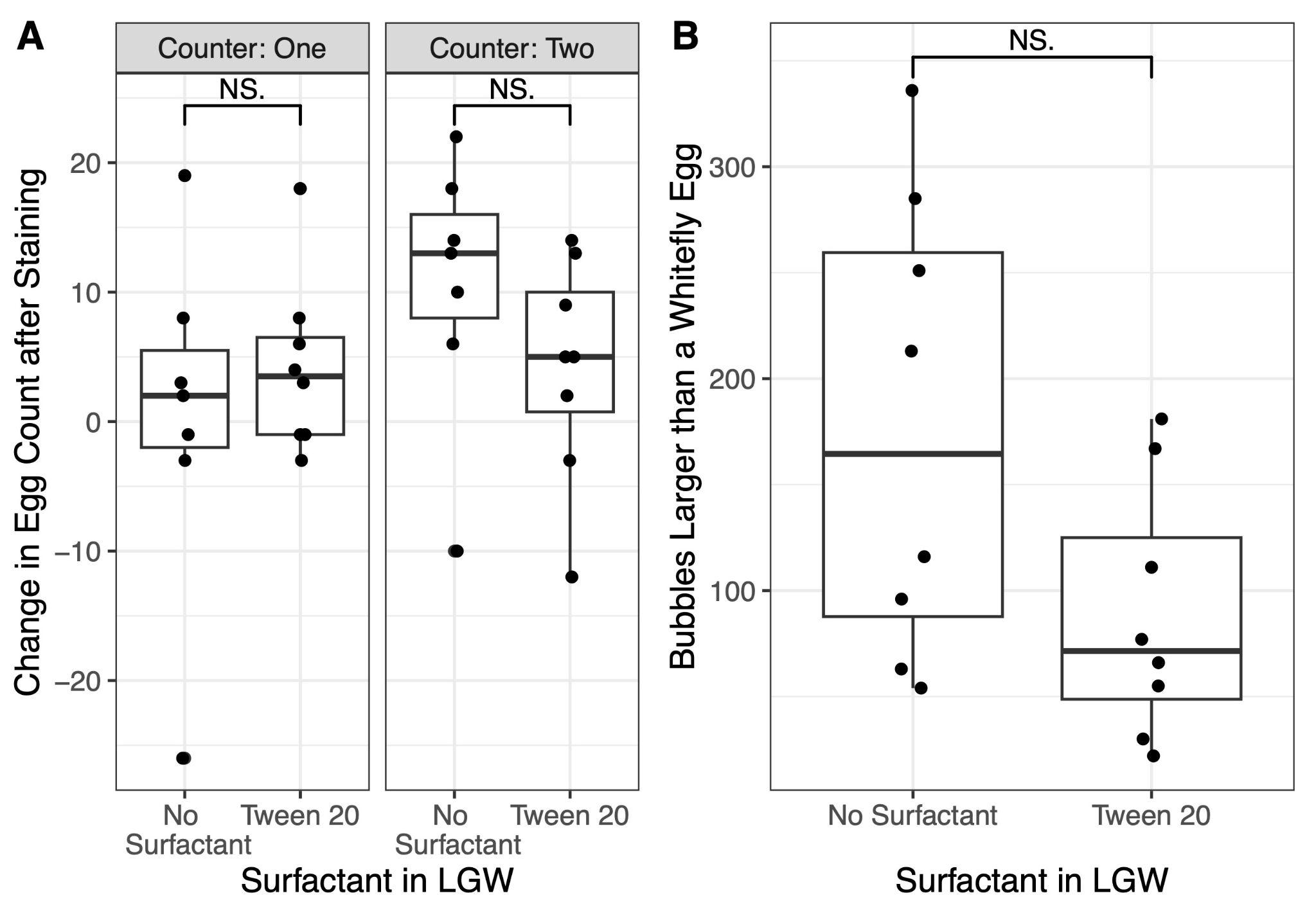


**Figure S4**. Tomato leaf disc response to clearing with and without 0.1 % (v/v) Tween 20 surfactant in the LGW solution. **A**. Change in egg count after the staining process for each counting individual. **B**. Number of bubbles on stained leaf discs.


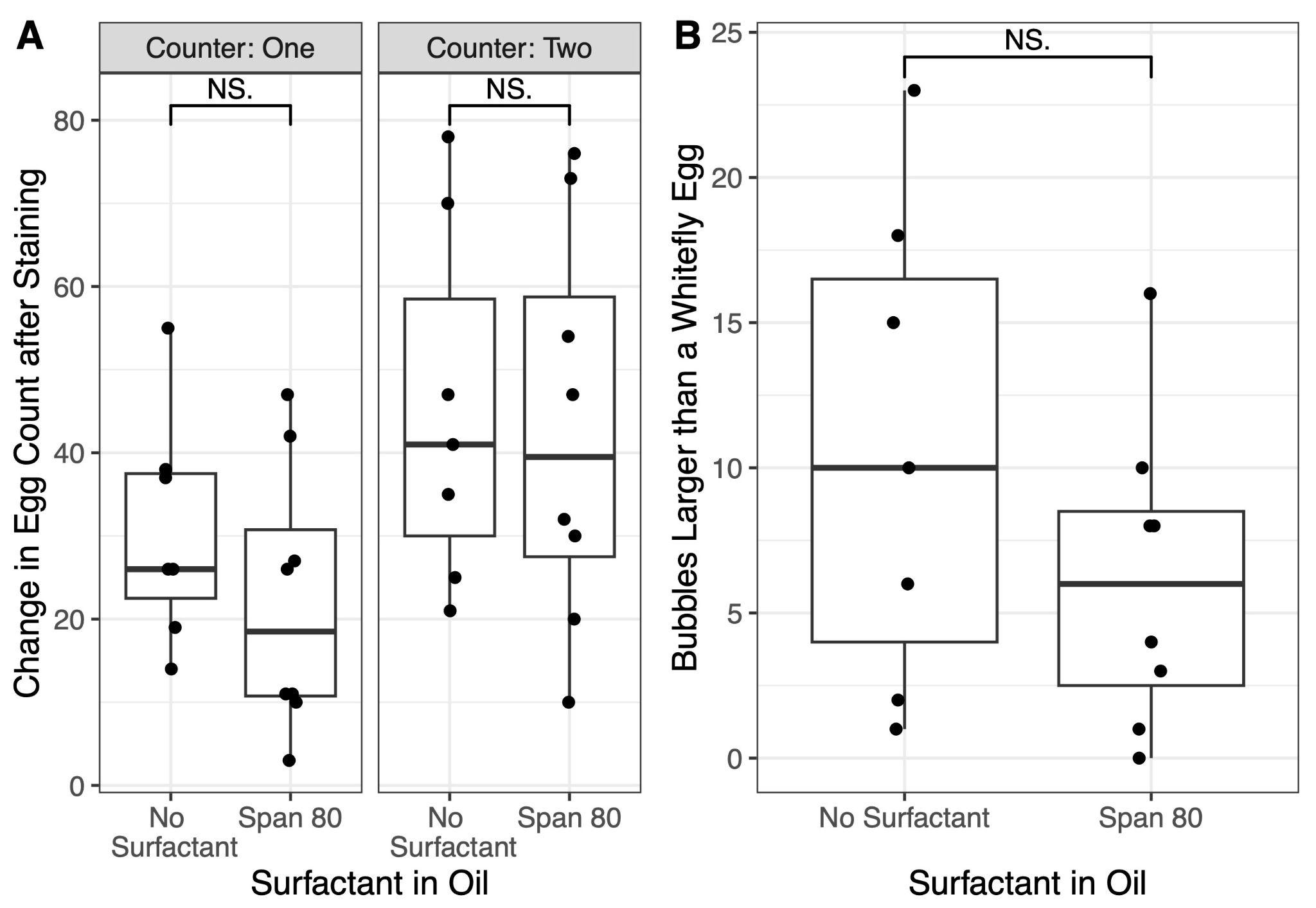


**Figure S5**. Melon leaf disc response to counting eggs in mineral oil with and without 0.1% (v/v) Span 80. **A**. Change in egg count after the staining process for each counting individual. **B**. Number of bubbles on stained leaf discs.


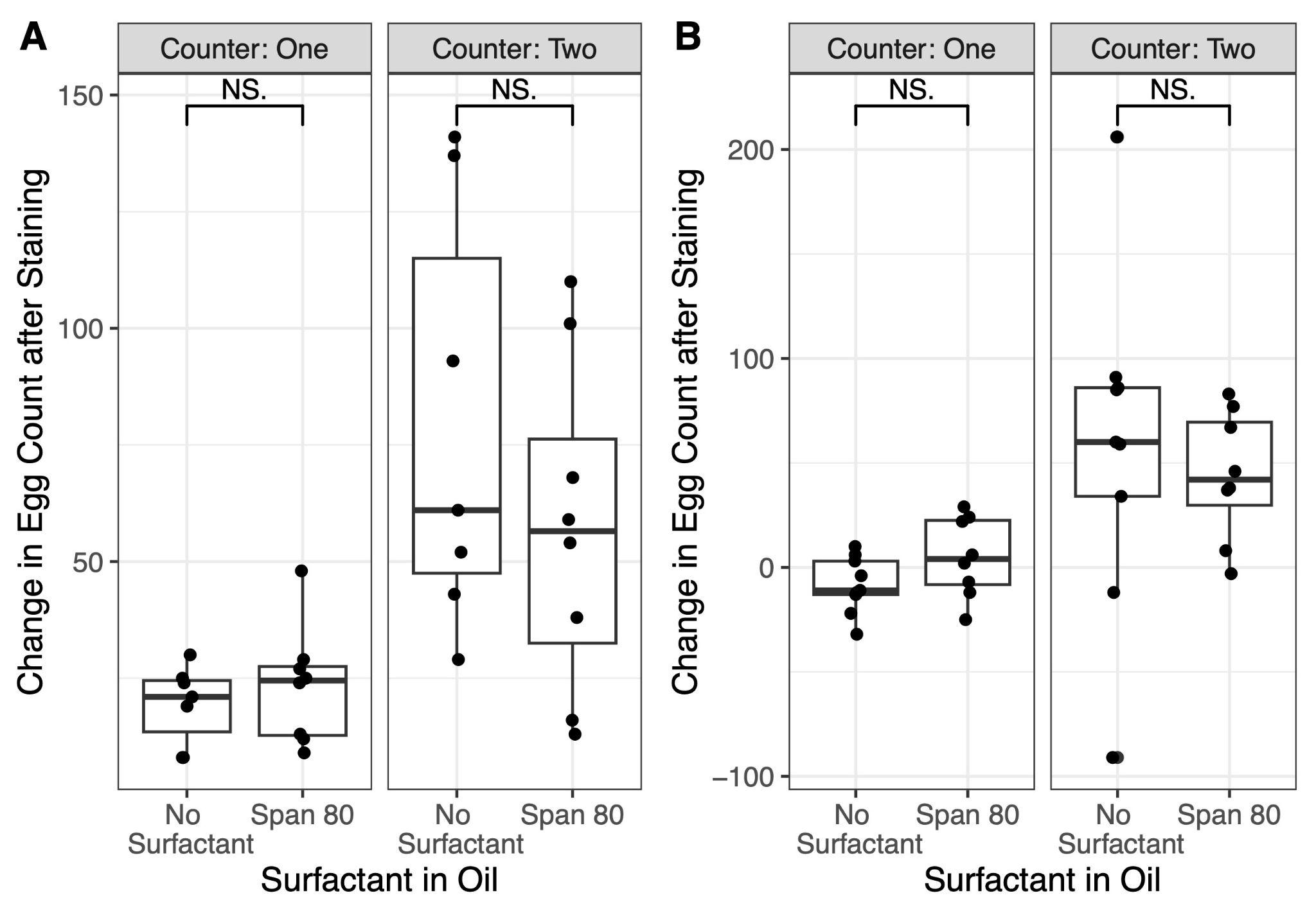


**Figure S6**. Change in egg count after the staining process for each counting individual when counted in mineral oil with and without 0.5% (v/v) Span 80. **A**. Cowpea. **B**. Sweet potato.


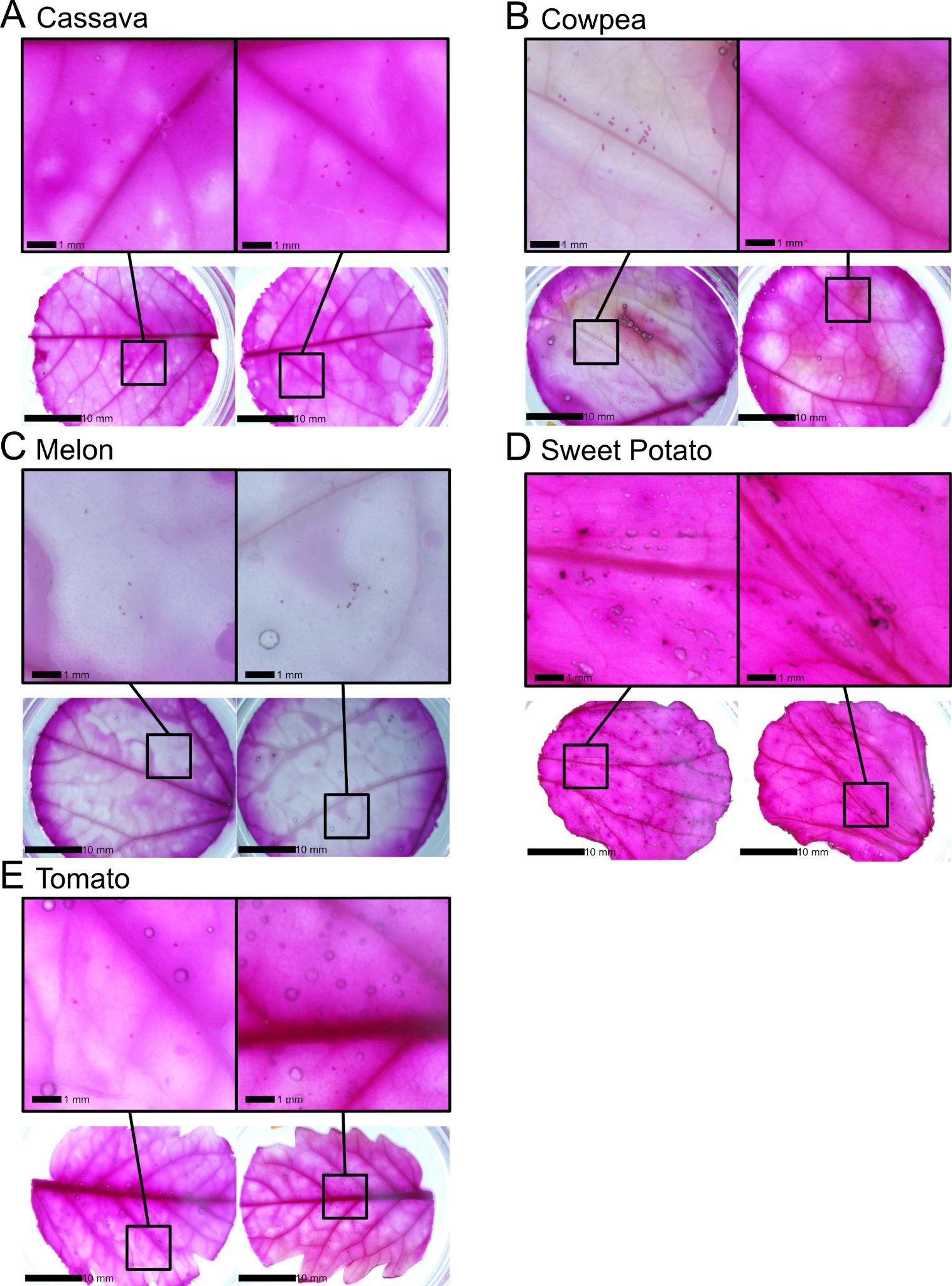


**Figure S7**. Stained images from each crop with the greatest (**left**) and least (**right**) increase in egg count after the staining process. These images provide an additional example of the possible outcomes of the staining process. Detail regions were selected to highlight why eggs might have been easy to see in the best images or hard to see in the worst images. **A**. Cassava. **B**. Cowpea. **C**. Melon. **D**. Sweet potato. **E**. Tomato.


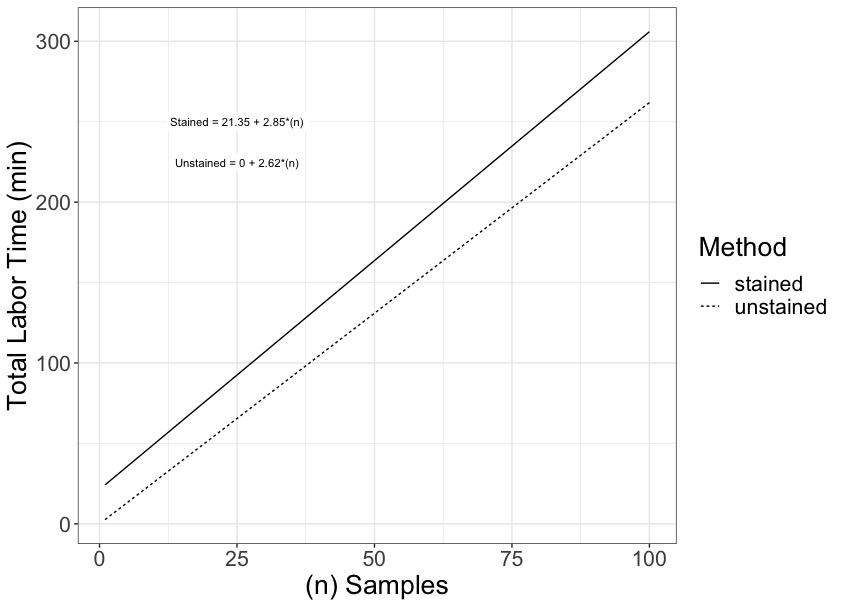


**Figure S8**. Comparison of per sample labor time requirements for obtaining stained and unstained egg counts including all setup and processing times.

#

# Supplemental Bibliography

[1. Backus EA, Hunter WB, Arne CN. Technique for staining leafhopper (Homoptera: Cicadellidae) salivary sheaths and eggs within unsectioned plant tissue. J Econ Entomol. 1988;81:1819–23.](http://paperpile.com/b/ozT3A8/JiJMe)

[2. Swenson VA, Stacy AD, Gaylor MO, Ushijima B, Philmus B, Cozy LM, et al. Assessment and verification of commercially available pressure cookers for laboratory sterilization. PLoS One. 2018;13:e0208769.](http://paperpile.com/b/ozT3A8/wmahO)
